# Pterostilbene and 3′-Hydroxypterostilbene Alleviate AOM/DSS-Induced and Chronic Jet Lag-Exacerbated Colon Cancer

**DOI:** 10.1021/acs.jafc.6c09704

**Published:** 2026-10-26

**Authors:** Yen-Chun Koh, Yen-Tung Chen, Pin-Yu Ho, Wei-Sheng Lin, Kai-Yu Hsu, Anju Majeed, Chi-Tang Ho, Min-Hsiung Pan

**Affiliations:** † Department of Seafood Science, 33561 National Kaohsiung University of Science and Technology, Kaohsiung 81157, Taiwan; ‡ Institute of Food Sciences and Technology, National Taiwan University, Taipei 10617, Taiwan; § Department of Food Science, 252857 National Quemoy University, Quemoy 89250, Taiwan; ∥ Sami-Sabinsa Group Limited, Bengaluru, Karnataka 560058, India; ⊥ Department of Food Science, 5970 Rutgers University, New Brunswick, New Jersey 08901, United States; # Department of Medical Research, China Medical University Hospital, China Medical University, Taichung City 40402, Taiwan; ∇ Department of Health and Nutrition Biotechnology, Asia University, Taichung City 41354, Taiwan

**Keywords:** Circadian disruption, colon cancer, pterostilbene, 3′-hydroxypterostilbene, β-catenin

## Abstract

Chronic circadian disruption is associated with colorectal cancer (CRC), but whether it exacerbates CRC progression and influences the protective effects of dietary interventions remains unclear. We examined pterostilbene (PSB) and 3′-hydroxypterostilbene (3′-HPSB) in an azoxymethane/dextran sulfate sodium-induced CRC mouse model exposed to chronic jet lag. Chronic jet lag aggravated disease activity, intestinal pathology, inflammation, and tumor burden. Dietary PSB and 3′-HPSB supplementations, initiated before CRC induction, attenuated these pathological changes and reduced macroscopic tumors (≥2 mm) by approximately 36% and 49%, respectively. These effects were accompanied by alterations in circadian clock-related and Wnt/β-catenin signaling-related proteins and by reduced intestinal epithelial proliferation. Supplementation was also associated with changes in gut microbiota composition and fecal short-chain fatty acid levels. These findings support preventive/protective effects of PSB and 3′-HPSB under chronic circadian disruption and identify associated molecular and microbial alterations warranting further mechanistic investigation.

## Graphical abstract

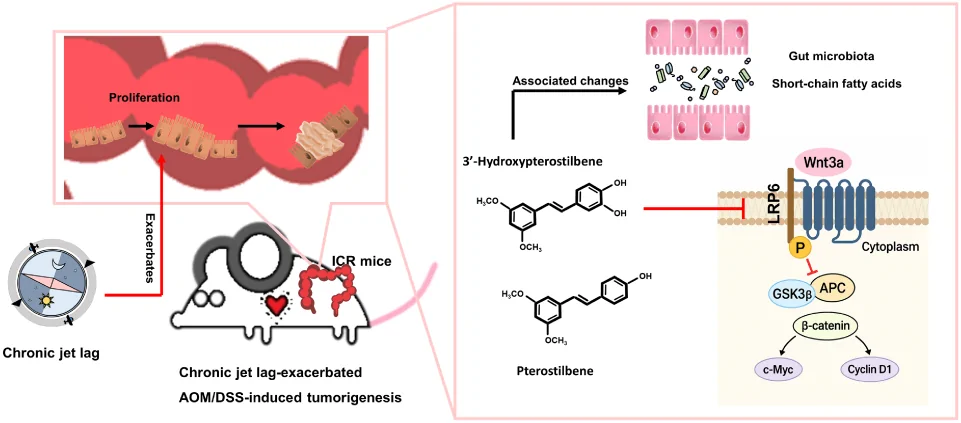

## Introduction

As one of the diseases often linked to a disrupted circadian rhythm, cancer has been widely studied for its association with the biological clock. The disruption of circadian clock genes by environmental or hormonal factors can lead to abnormal cell cycles, activation of inflammatory responses, and oncogenic signaling pathways.^1^ Extensive research has highlighted the relationship between circadian rhythm disorder (CRD) and tumorigenesis. For example, Hadadi et al. demonstrated that chronic jet lag promoted breast cancer cell proliferation and lung metastasis.^2^ Circadian clock genes regulate the expression of approximately 20% of genes in mammals, which are involved in various physiological responses, including cell cycle regulatory genes, oncogenes, and tumor suppressor genes. Therefore, disruptions to the circadian clock may impair normal cellular functions.^3^

Recent studies have highlighted a rapidly increasing trend in colorectal cancer (CRC) incidence among individuals aged <50 years, a phenomenon termed young-onset CRC (YO-CRC). Lifestyle-related risk factors, such as obesity, antibiotic overuse, lack of exercise, irregular eating habits, and exposure to artificial light at night, have been proposed as potential contributors to the increasing incidence of YO-CRC.^4^ As a tumor suppressor gene, clock circadian regulator (*CLOCK*) plays a crucial role in cellular responses to DNA damage. CLOCK mutations can alter these responses, promoting carcinogenesis. Additionally, *CLOCK* is prone to microsatellite instability (MSI), with over 50% of MSI-associated CRC cases having *CLOCK* mutations.^5^

The Wnt signaling pathway, one of the hallmark pathways in CRC, plays a pivotal role in its development. According to The Cancer Genome Atlas, nearly 67% of patients with sporadic CRC have mutations in the adenomatous polyposis coli (*APC*), 8% in ring finger protein 43 (*RNF43*), and 6% in catenin beta 1 (*CTNNB1*/β-catenin).^6^ The canonical Wnt pathway is aberrantly activated in over 90% of CRC cases.^7^ Recent studies have underscored the regulatory role of the canonical Wnt/β-catenin signaling pathway in CRC. The Wnt/β-catenin pathway is often activated by specific genetic mutations, highlighting its direct involvement in tumorigenesis.^8^ Whether through genetic or epigenetic mechanisms, aberrant Wnt/β-catenin signaling correlates positively with cancer development.

Regarding the TGF-β1-regulated pathway, previous studies have demonstrated the preventive potential of pterostilbene (PSB) in various diseases. In tumor protein p53 (TP53)-mutated breast cancer cells, PSB inhibited the expression of oncogenic proteins β-catenin and mechanistic target of rapamycin kinase (mTOR) while enhancing the expression of the pro-apoptotic protein BCL2 associated X, apoptosis regulator (BAX).^9^ In addition, Tolomeo et al. reported that PSB and 3′-hydroxypterostilbene (3′-HPSB) exhibited superior anticancer effects to resveratrol in leukemia cells.^10^ Notably, our previous study showed that 3′-HPSB inhibited interleukin 6 (IL-6) signaling, suppressed the expression of inflammatory enzymes, and reduced the activation of the β-catenin signaling pathway in a CRC mouse model.^11^

Circadian rhythm disruption has become a common issue in modern life, often associated with increased risks of chronic diseases, including cancer. The Wnt/β-catenin signaling pathway, a key driver of CRC development and progression, is excessively activated in over 90% of patients. This excessive activation not only promotes tumorigenesis but also poses challenges for treatment, as it reduces the efficacy of standard therapies. However, whether chronic circadian disruption further aggravates CRC progression and whether dietary interventions remain protective under such conditions require further investigation. Therefore, the present study aimed to determine (1) whether chronic jet lag exacerbates CRC progression in an AOM/DSS-induced CRC model and (2) whether dietary PSB and 3′-HPSB attenuate CRC progression under this disease-aggravating condition. We further characterized associated changes in circadian clock-related proteins, Wnt/β-catenin signaling, inflammatory responses, gut microbiota, and SCFAs to provide molecular context for the observed phenotypes.

## Materials and Methods

PSB and 3′-HPSB (purity > 98%) were provided by Sabinsa Corporation (NJ, USA). Primary antibodies used in this study were listed in Supporting Information.

### In Vivo Study Design

Male Institute of Cancer Research (ICR) mice were obtained from the BioLASCO Experimental Animal Center. The mice were housed under controlled conditions at 25 °C ± 1 °C and a relative humidity of 50% and maintained on a 12/12-h light/dark cycle. After a one-week acclimation period, the mice were randomly divided into five groups (*n* = 10/group): (1) control (CON), fed a normal diet and housed under a normal light/dark cycle; (2) LDAD, fed a normal diet, administered AOM/DSS, and housed under a normal light/dark cycle; (3) JAD, administered AOM/DSS and housed under a CRD light/dark cycle; (4) PSB, administered AOM/DSS, housed under a CRD light/dark cycle, and fed a diet supplemented with 0.025% PSB; and (5) 3′-HPSB, administered AOM/DSS, housed under a CRD light/dark cycle, and fed a diet supplemented with 0.025% 3′-HPSB Figure S1). The sample diets were administered 1 week prior as pretreatment, and the mice had ad libitum access to food and water. Following intraperitoneal injection of AOM, mice underwent two cycles of 2% DSS solution, each consisting of a seven-day treatment followed by a seven-day recovery with deionized water.

The dietary concentration of 0.025% 3′-HPSB was selected based on our previous studies demonstrating its protective effects in mouse models of intestinal inflammation.^12,13^ PSB was supplemented at the same concentration to enable direct comparison between PSB and 3′-HPSB under equivalent dietary conditions.

At the end of the study period, all mice were euthanized using a gradual-fill CO_2_ inhalation method, delivered at a displacement rate of 20–30% of the chamber volume per minute. Blood samples were collected via cardiac puncture and centrifuged to isolate the plasma. Liver function-related indicators in serum, including alanine aminotransferase (ALT) and aspartate aminotransferase (AST), were measured using a Hitachi 7080 automated blood biochemical analyzer. The organs were harvested and weighed, while the complete colons were carefully dissected from the cecum to the anus to collect fecal samples, which were stored at −80 °C for subsequent analysis. The intestines were then opened, placed on a scale plate for photographing, weighed, and measured for length, and the number of tumors of different sizes was counted. Part of the intestine was pinned to a wooden board using needles and fixed in formalin for later histological sectioning and staining. The remaining intestinal tissue was used for scraping intestinal proteins, which were stored at −80 °C for further analysis.

### Induction of Chronic Jet Lag

According to Chalfant et al. (2023),^14^ compared to a delayed light/dark cycle, it takes mice longer to resynchronize their internal rhythms with external cues under an advanced light/dark cycle, leading to more severe disruptions to their biological clock. They also found that it takes about 6 days for mice to readjust their activity and feeding patterns to align with the new light/dark cycle. Therefore, this study advanced the light period by 6 h every 5 days while maintaining a 12/12 light-dark cycle to disrupt the circadian rhythm in the mice.

### Disease Activity Measurement

During the DSS cycle, the disease activity index (DAI) of the mice was measured daily during DSS treatment and every other day during recovery with normal drinking water. After the DSS cycle ended, the DAI was measured weekly. Each indicator was measured and averaged daily. The area under the curve (AUC) was used to compare the overall disease activity between groups.

### Hematoxylin and Eosin Staining and Histopathological Analysis

Intestinal tissues (N = 3 per group) were fixed in 10% formalin buffer, dehydrated, and paraffin-embedded. Formalin-fixed paraffin-embedded tissues were sectioned at a thickness of 3–5 μm, deparaffinized in xylene, rehydrated through ethanol and water, and stained with hematoxylin and eosin (H&E). H&E-stained intestinal sections were submitted to the Laboratory Animal Center, College of Medicine, National Taiwan University, for histopathological evaluation. Histopathological changes were examined at 50× and 400× magnifications, and lesion severity was graded on a scale of 1–5. Histopathological scoring was performed in a blinded manner; slides were identified only by coded numbers, and the evaluator was unaware of the corresponding experimental group assignments.

### Proinflammatory Cytokine Analysis

Cytokine levels were determined in the supernatant of ex vivo cultured colon biopsies. First, the colon tissue was washed with phosphate-buffered saline containing antibiotics, immersed in a 24-well dish containing RPMI 1640 medium, and incubated at 37 °C for 24 h. After incubation, the culture medium was collected into a microcentrifuge tube and centrifuged at 14,000 × g for 1 h. The resulting supernatant was used in enzyme-linked immunosorbent assays. The list of cytokine ELISA kits was provided in Supporting Information. Due to occasional contamination during ex vivo culture, eight valid samples per group were randomly selected for cytokine analysis.

### Protein Analysis

The target protein expressions in liver, colon and tumor were determined by conducting Western blot (procedure is provided in Supporting Information). For intergroup comparisons, the normalized values were further normalized to the mean value of the CON or LDAD group, and the results are expressed as relative expression levels. Data are presented as mean ± SEM from four independent biological replicates (N = 4).

### Short-Chain Fatty Acid Analysis

The detail of analysis method of the levels of fecal short-chain fatty acids (SCFAs) was provided in Supporting Information.

### Gut Microbiota Analysis

Colonic fecal samples were collected and stored at −80 °C until analysis. The detail of analysis method was provided in Supporting Information.

### Statistical Analysis

Statistical analyses were performed using IBM SPSS Statistics version 22 (IBM Corp., Armonk, NY, USA). The variables are presented as mean ± standard error of mean (SEM). Differences among multiple groups were analyzed using one-way ANOVA followed by Tukey’s HSD post hoc test, whereas specific pairwise comparisons between two groups were analyzed using Student’s *t* test. A p-value <0.05 was considered statistically significant. Statistical outliers were identified using the ROUT method in GraphPad Prism and excluded prior to analysis when applicable.

## Results

### Physiological Monitoring of Mice under Experimental Conditions

Figure 1A shows the appearance of the mice, while Figure 1B illustrates body weight changes throughout the 13-week experimental period. All groups gained approximately 20 g during the study, with no significant differences in initial body weight, final body weight, or total weight gain. During the DSS treatment cycles, all AOM/DSS-treated groups exhibited transient body weight loss, which rapidly recovered after the resumption of normal drinking water (Figure 1C). Mean daily food and water intake are shown in Figure 1D. Based on food consumption, the estimated intake of PSB and 3′-HPSB was approximately 2.5 mg/day, corresponding to an estimated dose of 56.8 mg/kg body weight/day based on a mean body weight of 44 g.

**Figure 1:**
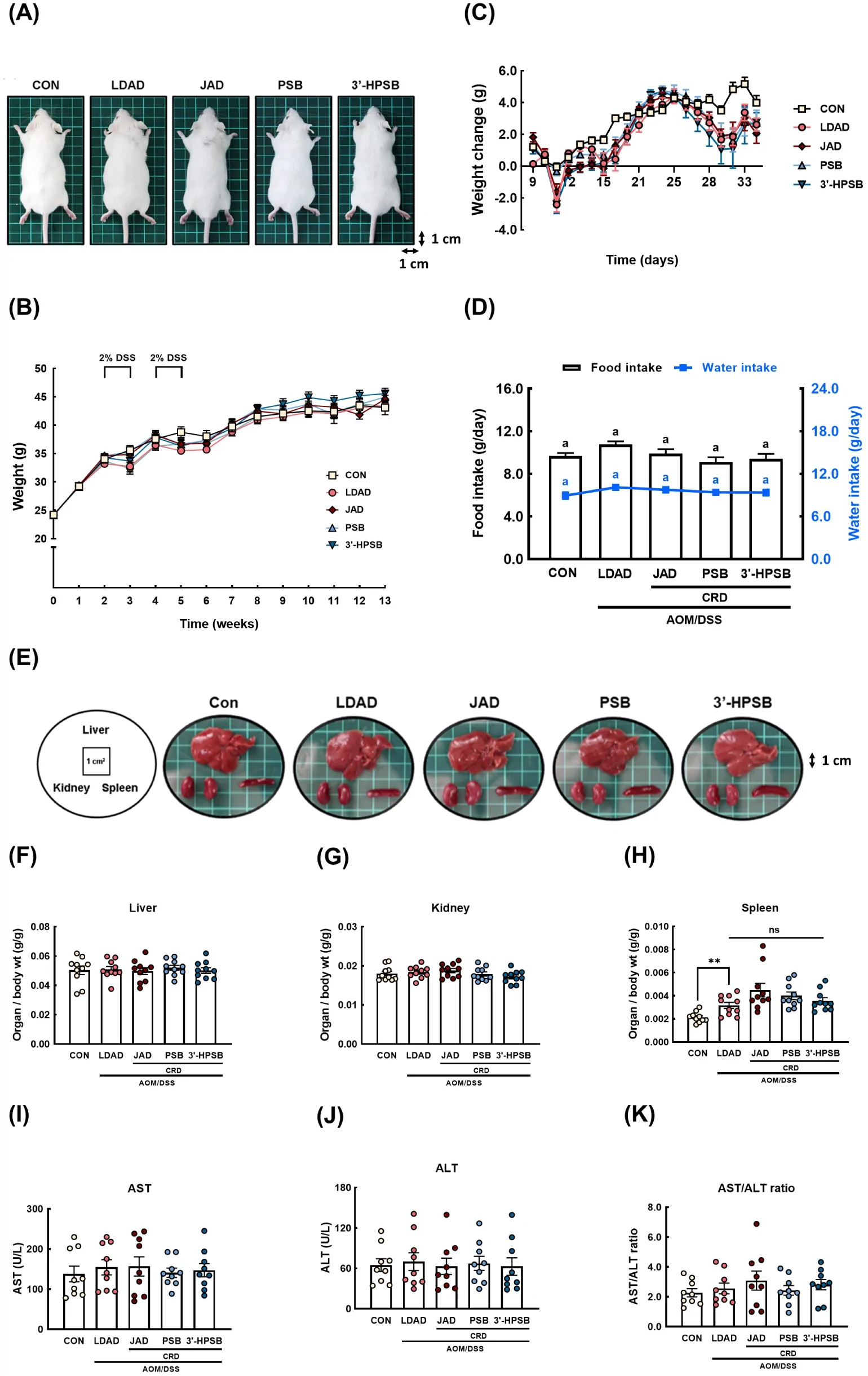
Physiological monitoring of mice under experimental conditions. Representative images of mice from each group. (B–C) Changes in body weight during the experiment. (D) Average food and water intake. (E) Representative images of the liver, kidney, and spleen from each group. Average organ indices of the (F) liver, (G) kidney, and (H) spleen. Serum levels of (I) aspartate transaminase (AST) and (J) alanine transaminase (ALT) and (K) the AST/ALT ratio. Data are presented as mean ± SEM from ten independent biological replicates (*N* = 9–10), and individual dots represent each replicate. Different lowercase letters indicate significant differences among groups, as determined by one-way ANOVA followed by posthoc Tukey’s HSD tests. ** indicates a significant difference between the compared groups at *p* < 0.01, as determined by Student’s *t* test. CON, control group; LDAD, AOM/DSS-treated group under a normal light-dark cycle; JAD, AOM/DSS-treated group exposed to chronic jet lag; PSB, JAD group supplemented with pterostilbene; 3′-HPSB, JAD group supplemented with 3′-hydroxypterostilbene.

Relative liver and kidney weights did not differ significantly among the groups (Figure 1F–G). Relative spleen weight was significantly higher in the LDAD group than in the CON group but did not differ among the AOM/DSS-treated groups (Figure 1H), suggesting that splenomegaly was primarily associated with DSS-induced inflammation. Similarly, no significant differences were observed in serum ALT or AST levels among the groups (Figure 1I–K).

### PSB and 3′-HPSB Significantly Reduce Intestinal Shortening, Weight-to-Length Ratio, and Tumor Formation

Figure 2A shows the changes in DAI scores throughout the 13-week experimental period, while Figure 2B illustrates DAI scores during the two DSS treatment cycles. These data were further summarized as the AUC of DAI scores (Figure 2C). The AUC increased significantly in the LDAD group compared with the CON group and was further elevated in the JAD group. In contrast, both PSB and 3′-HPSB treatments significantly reduced the DAI AUC relative to the JAD group. Representative colon images are shown in Figure 2D. Compared with the CON group, the LDAD group exhibited significantly shorter intestines, accompanied by increased intestinal weight and weight-to-length ratio (Figure 2E–G). These alterations were further aggravated in the JAD group but were significantly attenuated by PSB and 3′-HPSB treatment, suggesting protection against DSS-induced intestinal injury.

**Figure 2:**
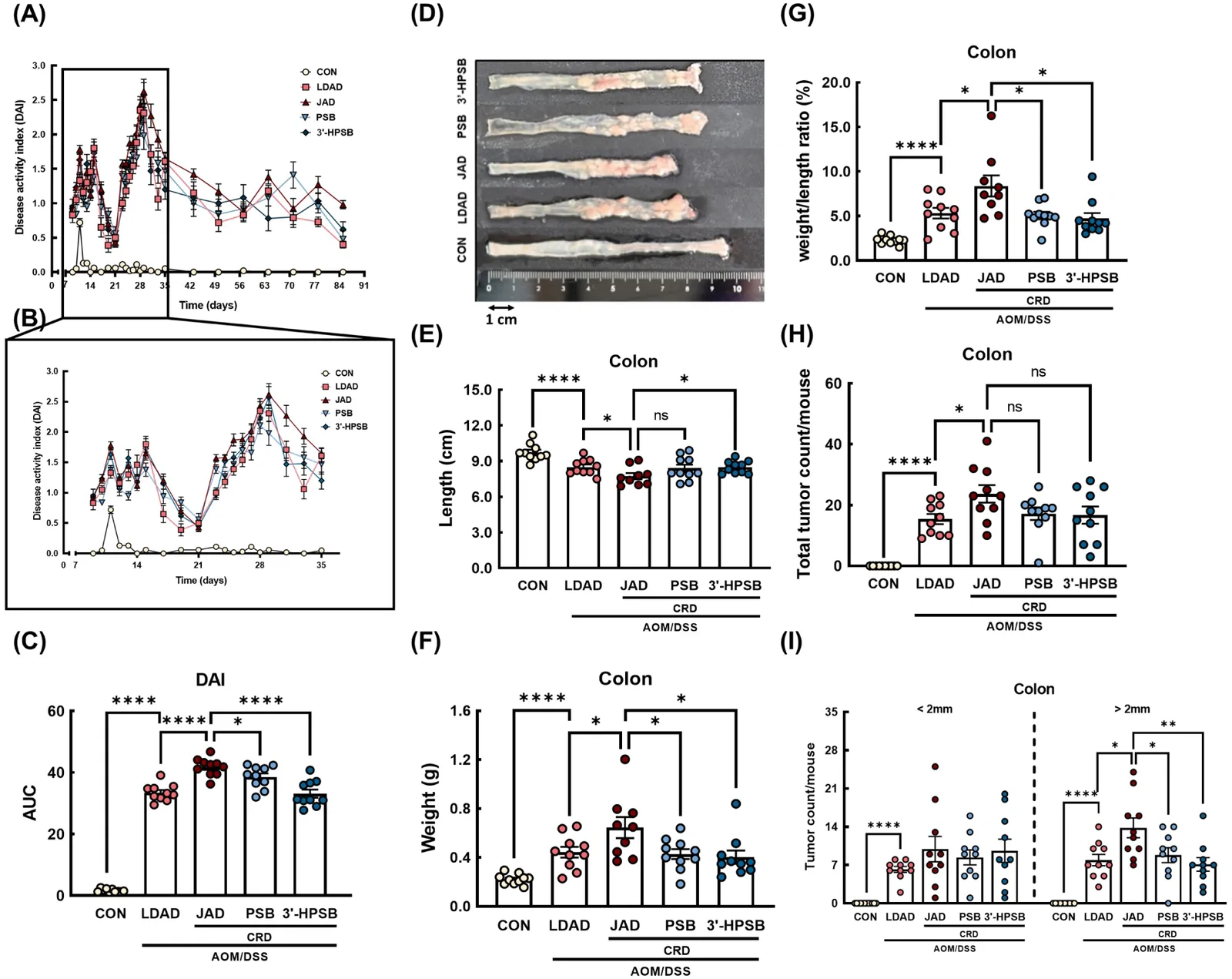
PSB and 3′-HPSB significantly reduce intestinal shortening, weight-to-length ratio, and tumor formation. The disease activity index (DAI) scores (A) throughout the experiment and (B) during the two DSS treatment cycles. (C) The area under curves (AUCs) calculated from (B). (D) Representative images of the colon from each group. (E) Colon length, (F) weight, and (G) weight-to-length ratio (%). (H) Total tumor count per mouse, and (I) the count of tumors smaller than 2 mm and larger than 2 mm. Data are presented as mean ± SEM from eight independent biological replicates (*N* = 9–10), and individual dots represent each replicate. *, **, ***, and **** indicate significant differences between the compared groups at *p* < 0.05, 0.01, 0.005, and 0.001, respectively, determined by Student’s *t* test. CON, control group; LDAD, AOM/DSS-treated group under a normal light-dark cycle; JAD, AOM/DSS-treated group exposed to chronic jet lag; PSB, JAD group supplemented with pterostilbene; 3′-HPSB, JAD group supplemented with 3′-hydroxypterostilbene.

The total number of intestinal tumors was significantly higher in the JAD group than in the LDAD group (Figure 2H). Tumor size analysis revealed no significant differences in tumors smaller than 2 mm among the experimental groups, and no such tumors were observed in the CON group (Figure 2I). In contrast, the number of tumors larger than 2 mm was significantly increased in the JAD group and significantly reduced by both PSB and 3′-HPSB supplementations. Consistent with the reduced tumor burden, representative immunofluorescence staining of tumor tissues qualitatively showed fewer *K*_i_-67-positive cells in the PSB- and 3′-HPSB groups than in the JAD group, suggesting reduced proliferative activity (Figure S2). Representative caspase-3 immunofluorescence staining also showed greater caspase-3 immunoreactivity in the PSB- and 3′-HPSB groups than in the JAD group (Figure S3).

### PSB and 3′-HPSB Attenuate AOM/DSS-Induced Colonic Inflammation, Tissue Damage, and Tumor Formation

Intestinal structural changes were evaluated using hematoxylin and eosin (H&E)-stained sections (Figure 3A). The CON group exhibited an intact intestinal architecture with orderly villi, minimal tissue damage, and no evidence of polyps or adenomas. In contrast, the LDAD group displayed disrupted crypt architecture, mucosal hyperplasia, and tubular adenoma formation, consistent with midstage CRC. These abnormalities were further aggravated in the JAD group, which exhibited extensive villus loss and larger tubular adenomas. Although crypt damage and structural abnormalities were still evident in the PSB and 3′-HPSB groups, tissue disorganization was less severe than that in the JAD group, and only early stage precancerous lesions were observed. Histopathological assessment was performed based on inflammation, hyperplasia, granulation tissue formation, and goblet cell depletion (Figure 3B–G). Compared with the CON group, all experimental groups showed significantly higher scores for inflammation, granulation tissue formation, goblet cell depletion, and hyperplasia. Among the intervention groups, intestinal hyperplasia was significantly reduced in the 3′-HPSB group. When the four pathological parameters were combined into a composite score, the JAD group exhibited significantly greater pathology than the LDAD group, whereas the 3′-HPSB group showed a significant reduction compared with the JAD group. No significant difference was observed in the PSB group.

**Figure 3:**
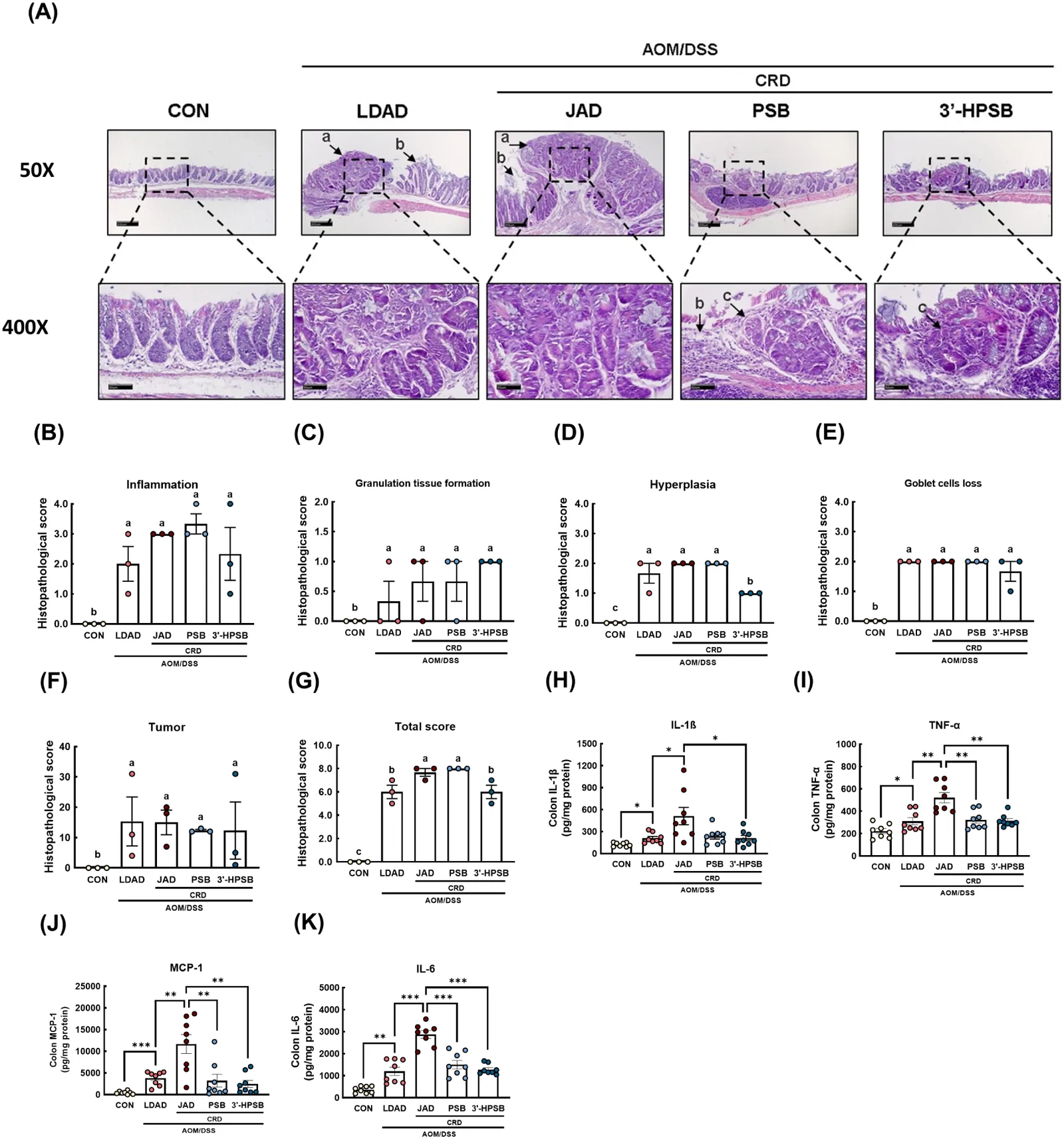
PSB and 3′-HPSB alleviate AOM/DSS-induced colonic inflammation, tissue damage, and tumor formation. (A) Representative images of H&E-stained colon and tumor sections. Histological scores for colonic (B) inflammation, (C) granulation tissue formation, (D) hyperplasia, (E) goblet cell loss, (F) tumors, and (G) the average total scores. Data are presented as mean ± SEM from three independent biological replicates (*N* = 3), and individual dots represent each replicate. The levels of colonic cytokines and chemokines (H) IL-1β, (I) TNF-α, (J) MCP-1, and (K) IL-6. Data are presented as mean ± SEM from eight independent biological replicates (*N* = 8), and individual dots represent each replicate. Different lowercase letters indicate significant differences among groups, as determined by one-way ANOVA followed by posthoc Tukey’s HSD tests. *, **, ***, and **** indicate significant differences between the compared groups at *p* < 0.05, 0.01, 0.005, and 0.001, respectively, determined by Student’s *t* test. CON, control group; LDAD, AOM/DSS-treated group under a normal light-dark cycle; JAD, AOM/DSS-treated group exposed to chronic jet lag; PSB, JAD group supplemented with pterostilbene; 3′-HPSB, JAD group supplemented with 3′-hydroxypterostilbene.

Intestinal inflammation was further evaluated by measuring the levels of interleukin-1β (IL-1β), IL-6, tumor necrosis factor-alpha (TNF-α), and MCP-1 (Figure 3H–K). All four inflammatory markers were significantly elevated in the LDAD group compared with the CON group and were further increased in the JAD group, consistent with the histopathological findings. Relative to the JAD group, PSB treatment significantly reduced IL-6, TNF-α, and MCP-1 levels, whereas 3′-HPSB treatment significantly reduced all four inflammatory markers.

### Impact of Chronic Jet Lag on Circadian Clock-Related Proteins in Liver, Tumor, and Intestinal Tissues

The effects of chronic jet lag on various organs were investigated by measuring circadian clock-related protein levels in liver, tumor, and intestinal tissues (Figure 4). In liver tissue, the levels of the positive regulatory protein BMAL1 were significantly lower in the JAD group than in the LDAD group but did not differ significantly between the PSB and 3′-HPSB groups and the JAD group. In contrast, the levels of other regulatory proteins did not differ significantly among the groups (Figure 4C–E).

**Figure 4:**
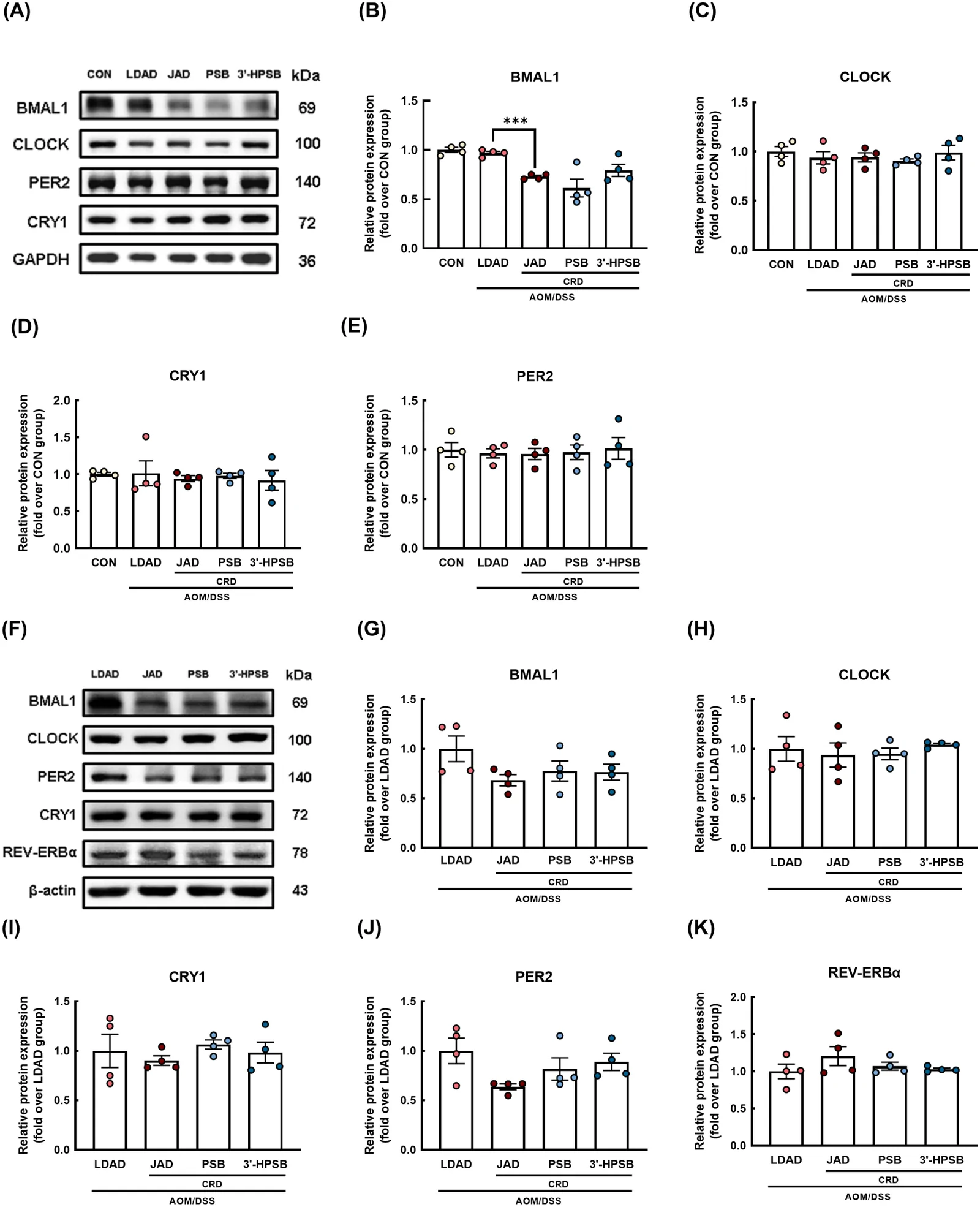
Impact of chronic jet lag on circadian clock protein levels in liver and tumor tissues. (A) Representative Western blot images and quantification of hepatic levels of (B) BMAL1, (C) CLOCK, (D) CRY1, and (E) PER2. (F) Representative Western blot images and quantification of tumor levels of (G) BMAL1, (H) CLOCK, (I) CRY1, (J) PER2, and (K) REV-ERBα. Data are presented as mean ± SEM from four independent biological replicates (*N* = 4), and individual dots represent each replicate. *** indicates significant differences between the compared groups at *p* < 0.005, determined by Student’s *t* test. Because the CON group did not undergo AOM/DSS induction and therefore did not develop tumors, tumor tissues were unavailable for Western blot analysis in this group. CON, control group; LDAD, AOM/DSS-treated group under a normal light-dark cycle; JAD, AOM/DSS-treated group exposed to chronic jet lag; PSB, JAD group supplemented with pterostilbene; 3′-HPSB, JAD group supplemented with 3′-hydroxypterostilbene.

In tumor tissue, the levels of the positive regulatory protein BMAL1 were lower in the JAD group than in the LDAD group but higher in the PSB and 3′-HPSB groups than in the JAD group (Figure 4F, G). However, no significant changes in CLOCK, cryptochrome circadian regulator 1 (CRY1), PER2, and reverse eukaryotic regulatory binding protein alpha (REV-ERBα) levels were observed (Figure 4H–K).

In intestinal tissue, the levels of the positive regulatory protein BMAL1 were significantly lower in the JAD group than in the LDAD group (Figure 5B). In contrast, they were higher in the PSB and 3′-HPSB groups than in the JAD group. The levels of another positive regulatory protein, CLOCK, were significantly lower in the JAD group than in the LDAD group (Figure 5C). In contrast, they were significantly higher in the PSB and 3′-HPSB groups than in the JAD group. The levels of negative regulatory protein PER2 were significantly lower in the LDAD group than in the CON group (Figure 5E). They were also significantly lower in the JAD group than in the LDAD group. Moreover, they were higher in the PSB and 3′-HPSB groups than in the JAD group. The levels of the negative regulatory protein CRY1 only showed an upward trend in the JAD group and did not differ significantly among the other groups (Figure 5D). Finally, the levels of the auxiliary negative regulatory protein REV-ERBα were significantly higher in the JAD group than in the LDAD group (Figure 5F). In contrast, they were higher in the PSB and 3′-HPSB groups than in the JAD group.

**Figure 5:**
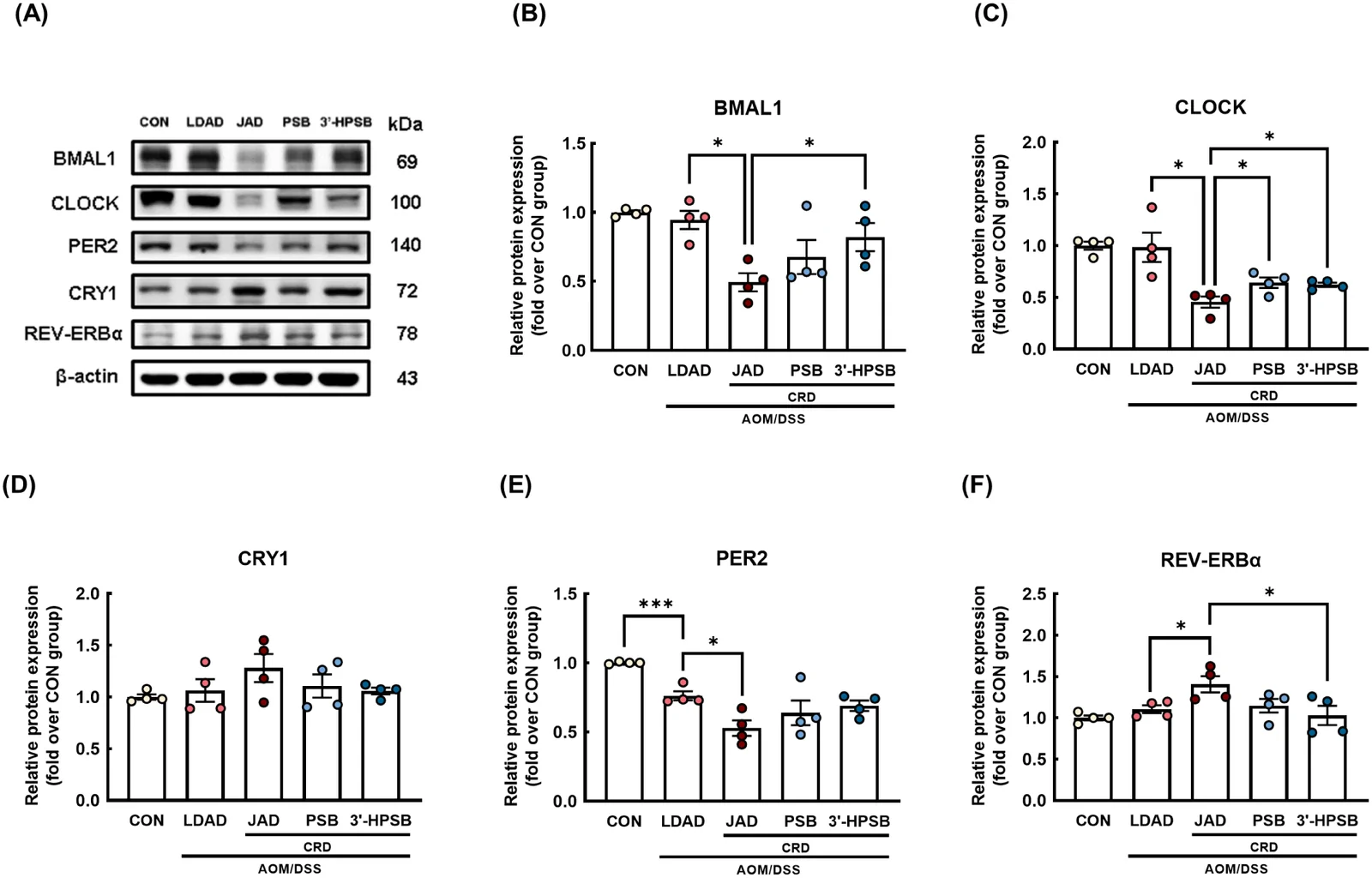
Impact of chronic jet lag on circadian clock protein levels in the colonic mucosa. (A) Representative Western blot images and quantification of colonic levels of (B) BMAL1, (C) CLOCK, (D) CRY1, (E) PER2, and (F) REV-ERBα. Data are presented as mean ± standard error of the mean (SEM) from four independent biological replicates (*N* = 4), and individual dots represent each replicate. *, **, ***, and **** indicate significant differences between the compared groups at *p* < 0.05, *p* < 0.01, *p* < 0.005, and *p* < 0.001, respectively, as determined by Student’s *t* test. CON, control group; LDAD, AOM/DSS-treated group under a normal light-dark cycle; JAD, AOM/DSS-treated group exposed to chronic jet lag; PSB, JAD group supplemented with pterostilbene; 3′-HPSB, JAD group supplemented with 3′-hydroxypterostilbene.

### Effects of PSB and 3′-HPSB on Wnt/β-Catenin Signaling-Related Proteins in Colonic and Tumor Tissues

In the intestine, wingless-type MMTV integration site family, member 3A (Wnt3A) levels showed an increasing trend in the JAD group but did not differ significantly among groups (Figure 6A–B). In contrast, phosphorylated low-density lipoprotein receptor-related protein 6 (p-LRP6) levels were elevated in the LDAD group compared with the CON group and were further increased in the JAD group. PSB treatment showed a tendency to reduce p-LRP6 levels, whereas 3′-HPSB significantly reduced p-LRP6 levels compared with the JAD group. Proteins associated with the β-catenin destruction complex, including APC, glycogen synthase kinase-3 beta (GSK3β), and axis inhibition protein 1 (AXIN1), were reduced in the LDAD group relative to the CON group (Figure 6D-H). APC and GSK3β levels were further decreased in the JAD group but were restored by both PSB and 3′-HPSB treatment, whereas AXIN1 levels remained unchanged. Consistent with these findings, β-catenin levels increased progressively from the LDAD to JAD groups and were significantly reduced by both interventions. Consistent with the Western blot findings, representative immunofluorescence staining of intestinal epithelial regions qualitatively showed greater β-catenin immunoreactivity in the JAD group than in the LDAD group, whereas lower β-catenin immunoreactivity was observed in the PSB and 3′-HPSB groups than in the JAD group (Figure S4). Similarly, c-Myc expression was elevated in the LDAD group and further increased in the JAD group, while PSB and 3′-HPSB significantly suppressed its expression. CCND1 levels were increased in the JAD group and showed a downward trend following PSB and 3′-HPSB treatment.

**Figure 6:**
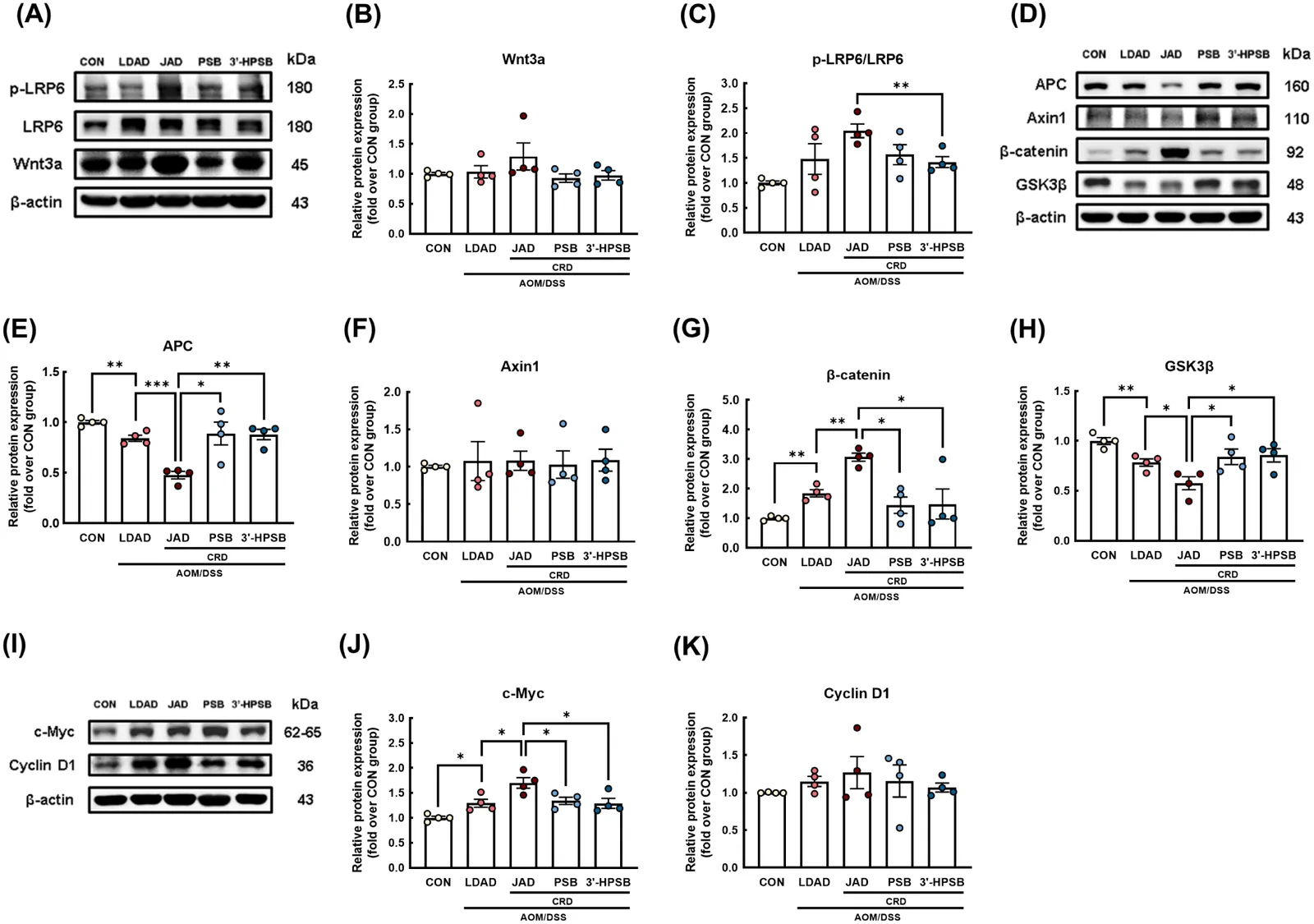
Effects of PSB and 3′-HPSB on Wnt/β-catenin signaling-related proteins in colonic tissue. (A) Representative Western blot images and quantification of colonic levels of (B) Wnt3A, (C) p-LRP6/LRP6, and (D) APC. (E) Representative Western blot images and quantification of colonic levels of (F) AXIN1, (G) β-catenin, and (H) GSK3β. (I) Representative Western blot images and quantification of colonic levels of (J) c-Myc and (K) cyclin D1. Data are presented as mean ± SEM from four independent biological replicates (*N* = 4), and individual dots represent each replicate. *, **, ***, and **** indicate significant differences between the compared groups at *p* < 0.05, *p* < 0.01, *p* < 0.005, and *p* < 0.001, respectively, as determined by Student’s *t* test. CON, control group; LDAD, AOM/DSS-treated group under a normal light-dark cycle; JAD, AOM/DSS-treated group exposed to chronic jet lag; PSB, JAD group supplemented with pterostilbene; 3′-HPSB, JAD group supplemented with 3′-hydroxypterostilbene.

In tumor tissues, WNT3A expression did not differ among groups (Figure 7). Although p-LRP6 levels were significantly higher in the JAD group than in the LDAD group, neither PSB nor 3′-HPSB reduced p-LRP6 levels. Similarly, no significant differences were observed in APC, GSK3β, AXIN1, β-catenin, or cyclin D levels. c-Myc expression was significantly increased in the JAD group compared with the LDAD group, but neither intervention improved this alteration.

**Figure 7:**
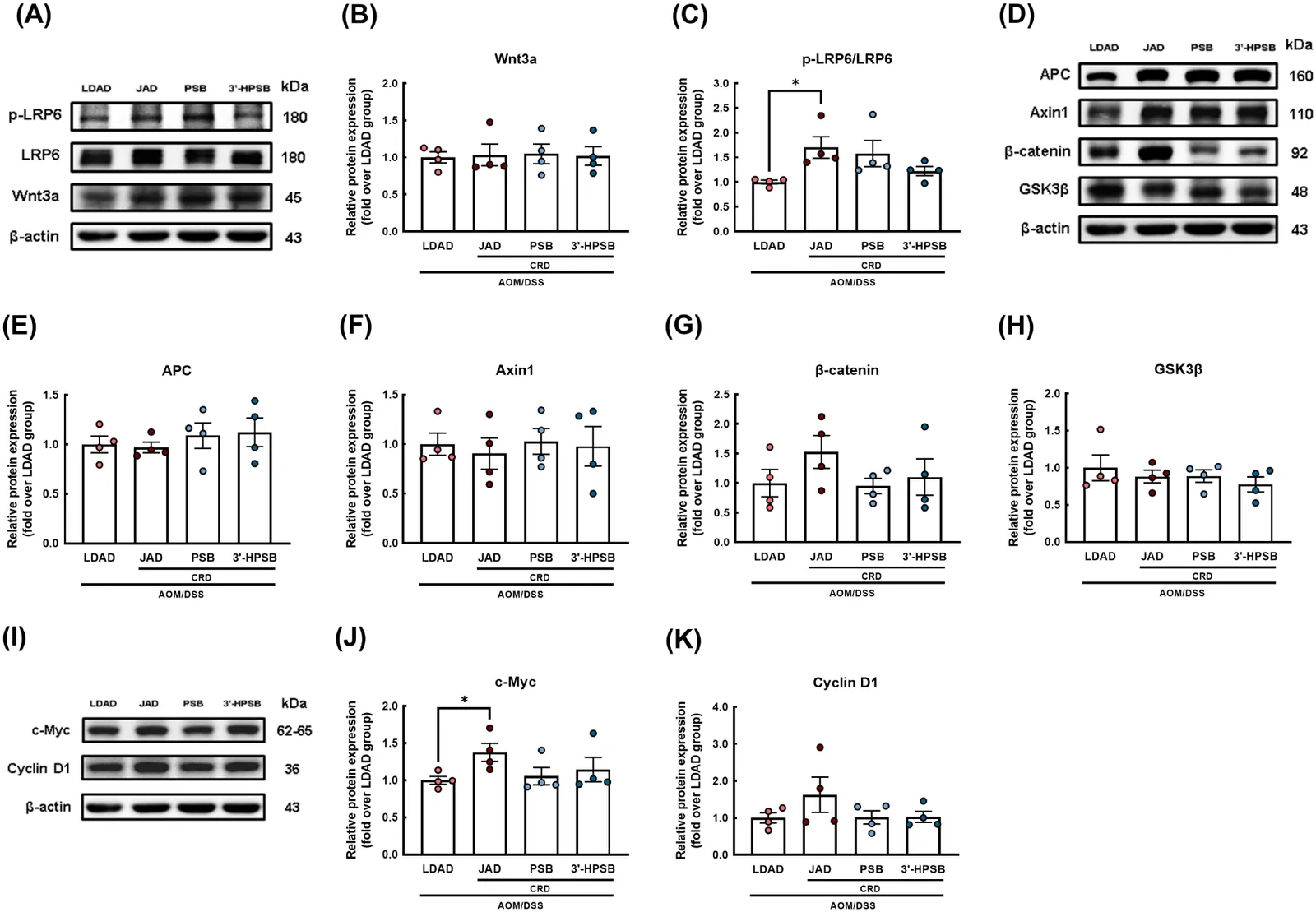
Effects of PSB and 3′-HPSB on Wnt/β-catenin signaling-related proteins in tumor tissue. (A) Representative Western blot images and quantification of tumor levels of (B) Wnt3A, (C) p-LRP6/LRP6, and (D) APC. (E) Representative Western blot images and quantification of tumor levels of (F) AXIN1, (G)­β-catenin, and (H) GSK3β. (I) Representative Western blot images and quantification of tumor levels of (J) c-Myc and (K) cyclin D1. Data are presented as mean ± SEM from four independent biological replicates (*N* = 4), and individual dots represent each replicate. * indicates significant differences between the compared groups at *p* < 0.05, as determined by Student’s *t* test. Because the CON group did not undergo AOM/DSS induction and therefore did not develop tumors, tumor tissues were unavailable for Western blot analysis in this group. CON, control group; LDAD, AOM/DSS-treated group under a normal light-dark cycle; JAD, AOM/DSS-treated group exposed to chronic jet lag; PSB, JAD group supplemented with pterostilbene; 3′-HPSB, JAD group supplemented with 3′-hydroxypterostilbene.

### Gut Microbial Modulation and SCFA Production May Partially Contribute to the Attenuation of CRC Progression

Intestinal acetate and butyrate levels were significantly lower in the LDAD group than in the CON group (Figure 8A–E). The JAD group exhibited further reductions in acetate, propionate, and butyrate compared with the LDAD group. In contrast, PSB supplementation significantly increased butyrate levels, while acetate and propionate showed increasing trends. Similarly, 3′-HPSB supplementation significantly increased acetate and butyrate levels, with a tendency toward increased propionate. These findings indicate that alterations in fecal SCFA levels occurred alongside the aggravated CRC phenotype under chronic jet lag and were partially reversed by PSB and 3′-HPSB supplementation.

**Figure 8:**
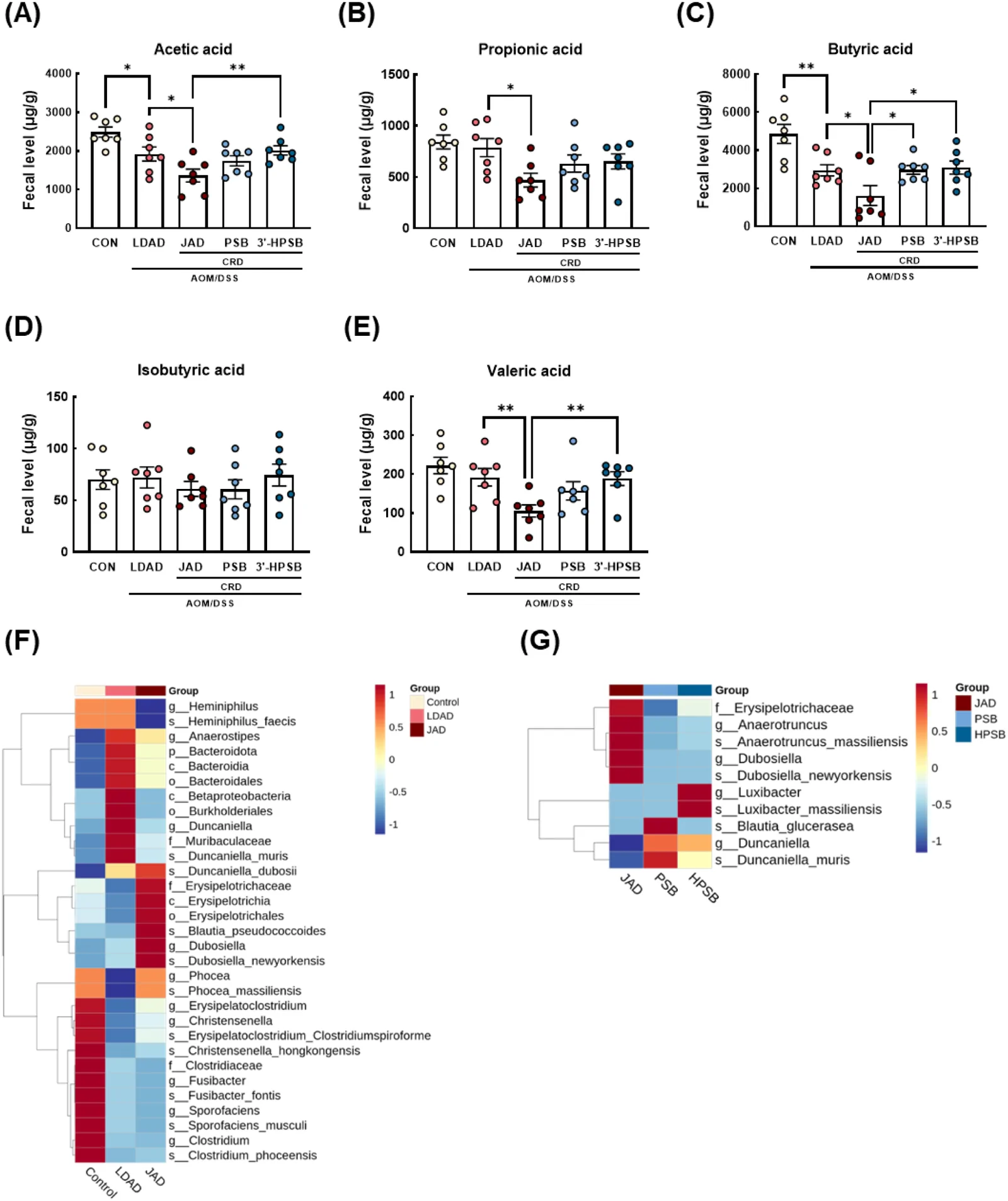
Alterations in gut microbial composition and SCFA levels among experimental groups. Fecal levels of SCFAs (A) acetic acid, (B) propionic acid, (C) butyric acid, (D) isobutyric acid, and (E) valeric acid. Data are presented as mean ± SEM from seven independent biological replicates (*N* = 7), and individual dots represent each replicate. (F–G) LefSe analysis among the compared groups. *, **, ***, and **** indicate significant differences between the compared groups at *p* < 0.05, 0.01, 0.005, and 0.001, respectively, determined by Student’s *t* test. CON, control group; LDAD, AOM/DSS-treated group under a normal light-dark cycle; JAD, AOM/DSS-treated group exposed to chronic jet lag; PSB, JAD group supplemented with pterostilbene; 3′-HPSB, JAD group supplemented with 3′-hydroxypterostilbene.

Mantel’s test (Figure S5) indicated a negative correlation between SCFA production and tumor number, particularly acetic acid, butyric acid, and valeric acid. Conversely, DAI scores were positively correlated with tumor numbers, suggesting that disease severity, as measured by DAI, increased alongside tumor burden.

No significant differences in α-diversity were observed (Figure S6), while the principal coordinates analysis (PCoA) and partial least-squares discriminant analysis (PLS-DA) plots (Figure S7A–B) suggested distinct gut microbial compositions between the JAD group and the other groups. These findings indicate that alterations in gut microbiota composition were associated with PSB and 3′-HPSB supplementation but do not establish a causal contribution of these microbial changes to the observed attenuation of CRC progression. In addition, the LEfSe analysis (Figure 8F–G) identified microbial taxa that differed significantly between groups. When microbial communities were compared among the CON, LDAD, and JAD groups at the genus level (Figure 8F), the CON group had a higher relative abundance of *Clostridium* and *Erysipelatoclostridium* compared to the LDAD and JAD groups. In contrast, the LDAD and JAD groups had a higher relative abundance of *Dubosiella*, with the LDAD group significantly surpassing the CON group (Figure S7). A comparison of microbial communities among the JAD, PSB, and 3′-HPSB groups at the genus level showed a significantly lower relative abundance of *Dubosiella* in the PSB and 3′-HPSB groups than in the JAD group (Figure 8G and S7).

## Discussion

Our study investigated whether chronic circadian rhythm disruption exacerbates AOM/DSS-induced CRC and whether dietary PSB and 3′-HPSB attenuate CRC progression under this disease-aggravating condition. Compared with the LDAD group, mice exposed to chronic jet lag exhibited greater disease activity, intestinal pathology, inflammation, and tumor burden, indicating that chronic jet lag aggravated the AOM/DSS-induced CRC phenotype. PSB and 3′-HPSB supplementations attenuated several of these pathological changes in the JAD model. These protective effects were accompanied by alterations in circadian clock-related protein levels and Wnt/β-catenin signaling-related proteins in intestinal tissue, together with decreased epithelial cell proliferation. These concurrent molecular changes provide context for the observed protective effects but do not establish that modulation of circadian rhythm mediates the effects of PSB or 3′-HPSB.

In this study, both PSB and 3′-HPSB supplementations significantly reduced the number of macroscopic tumors (≥2 mm) by approximately 36% and 49%, respectively, compared with the JAD group. Although the total tumor counts remained unchanged, the reduction in macroscopic lesions suggests that these compounds primarily suppress tumor progression rather than initiation. In the AOM/DSS colitis-associated colorectal cancer model, macroscopic tumors (≥2 mm) are widely recognized as clinically meaningful end points, as larger lesions are more likely to display high-grade dysplasia and progress toward invasive carcinoma.^15^ Several studies have similarly used ≥2 mm lesions to evaluate intervention efficacy and reported that reductions in macroscopic tumor burden correlate with decreased *K*_i_-67 labeling index and suppressed Wnt/β-catenin signaling, supporting the relevance of this end point to tumor progression.^16^ Moreover, while direct comparison with clinically used drugs using the same ≥2 mm end point is limited, analogous AOM/DSS studies have shown that 5-aminosalicylic acid (5-ASA) significantly reduces the incidence of >3 mm lesions with an approximate reduction of ∼50–60% based on reported tumor counts, highlighting that targeting macroscopic tumor burden is a relevant and translatable preventive strategy.^17^ Our findings suggest that PSB and 3′-HPSB may confer protective effects against CRC progression, warranting further evaluation in other preclinical models and early phase clinical studies.

Our study measured key proteins involved in the circadian clock transcription-translation feedback loop in the liver to characterize molecular alterations associated with the chronic jet lag protocol. Previous research has shown that when core circadian clock genes *Bmal1* or *Cry1*/2 are knocked out, the feeding rhythm of mice disappears.^18^ Additionally, *Bmal1* knockout leads to insulin resistance and liver fibrosis in mice,^19^ underscoring the critical role of BMAL1 in physiological liver functions. Our results showed a significant reduction in hepatic BMAL1 levels under the chronic jet lag condition. Zhang et al. found that disrupting the circadian rhythm in mice via sleep deprivation resulted in metabolic syndrome and significantly lower liver levels of BMAL1 in the circadian disruption group compared to the normal light-dark cycle group.^20^ Our findings also showed significant changes in core clock proteins in the colonic mucosa (Figure 5).

Clinical studies indicate that circadian rhythm disruption may accelerate CRC progression. Circadian clock-related genes are abnormally expressed in CRC tissues, suggesting that circadian clock proteins, particularly BMAL1, CLOCK, PER, and CRY, may influence the CRC course.^21,22^ Research shows that BMAL1 plays a crucial role in regulating tumor cell apoptosis, cell cycle progression, and DNA damage response, and it is considered a tumor suppressor gene. Its expression is reduced by nearly 50% in patients with CRC.^23^ The BMAL1/CLOCK complex also regulates the oncogene *c-Myc* during the G0/G1 phase transition.^24^ Moreover, in circadian rhythm-disrupted mice, PER2 levels in the intestine significantly decrease and lose their rhythmicity.^25^ Research also indicates that cell cycle protein p53 negatively regulates *PER2* expression, and PER2 is also known to regulate β-catenin and the cell cycle.^26^ Our results showed significantly lower levels of BMAL1, CLOCK, and PER, but significantly higher levels of REV-ERB in the colon of the JAD group. Previous studies have reported that REV-ERBα is highly expressed in patients with CRC,^27^ and that the tumor suppressor protein F-box and WD repeat domain containing 7 (FBXW7), in conjunction with cyclin-dependent kinase 1 (CDK1), mediates REV-ERBα degradation to regulate circadian rhythm.^28^ In addition, a recent study reported that pterostilbene (PSB) can bind to the coactivator protein RAR-related orphan receptor gamma (RORγ).^20^ As an auxiliary positive regulator of the circadian clock, RORγ can enhance Bmal1 expression. This previous finding provides a potential biological context for the alterations in clock-related proteins observed in the present study; however, whether PSB directly modulates RORγ or circadian clock function under our experimental conditions remains to be determined. It also remains possible that the observed changes in circadian clock-related proteins occurred secondarily to improvements in inflammation, tumor burden, or other disease-associated physiological alterations, rather than reflecting a direct effect of PSB or 3′-HPSB on the circadian machinery. Accordingly, the observed changes in circadian clock-related proteins should be interpreted as associated molecular alterations rather than evidence of a causal role in CRC progression.

Furthermore, recent studies have demonstrated that c-Myc can influence the circadian clock through multiple pathways. One mechanism involves c-Myc entering the nucleus and binding to the *REV-ERBα* promoter to promote its expression, whereas another involves c-Myc binding to the transcription factor Myc-interacting zinc finger protein 1 (MIZ1) and inhibiting *Bmal1* and *CLOCK* expression.^29^ Both mechanisms may indirectly reduce the levels of these essential circadian clock proteins. In line with this, our study showed significantly higher c-Myc levels in the JAD group compared with the LDAD group, suggesting a potential correlation between circadian clock dysregulation and c-Myc activation (Figure 6).

Given that β-catenin translocation into the nucleus initiates the expression of downstream oncogenic targets, including c-Myc, and that c-Myc is upregulated in CRC cells compared with normal intestinal mucosa,^30^ dysregulation of the Wnt/β-catenin pathway may play a central role in tumor development. In addition to its role in intestinal tumorigenesis, c-Myc has also been implicated in other CRC-related processes, including apoptosis, proliferation, metastasis, and chemotherapy resistance.^31^ Interestingly, the alterations in circadian clock-related proteins observed in the present study occurred alongside Wnt/β-catenin signaling-related proteins in the present model. Previous mechanistic evidence has demonstrated a link between circadian clock disruption and Wnt signaling in CRC. Specifically, Chun et al. showed that intestinal Bmal1 disruption accelerated Apc loss of heterozygosity, resulting in Wnt signaling hyperactivation and increased c-Myc expression in an Apc mutant CRC model.^32^ These findings provide a biological basis for considering whether the concurrent alterations observed in the present study may be related. However, because neither circadian clock components nor Wnt/β-catenin signaling were directly manipulated, our findings do not establish a causal or directional relationship between these pathways. Further mechanistic studies involving direct manipulation of circadian clock components and/or Wnt/β-catenin signaling will be required to determine the nature of this potential interaction.

A previous study found that PSB downregulated β-catenin and its downstream proteins, including cyclin D, c-Myc, and nuclear factor kappa B (NF-κB), suppressing colon tumorigenesis, cell proliferation, and inflammatory markers.^33^ A recent study also reported that PSB inhibited triple-negative breast cancer by suppressing epithelial-to-mesenchymal transition and stemness, mediated by β-catenin.^34^ Additionally, it was found that PSB isothiocyanate, a semisynthetic compound, could inhibit the proliferation of osteosarcoma cells by disrupting the interaction between β-catenin and transcription factor 4 (TCF-4). This disruption occurs through the formation of a stable complex with TCF-4, as demonstrated by molecular docking and luciferase assays.^35^ In comparison, only a few studies have reported the antiproliferative effect of 3′-HPSB in cancer research.^36^ In CRC cell lines COLO 205, HCT-116, and HT-29, 3′-HPSB was shown to activate caspase 3 (CASP3) and downregulate cyclooxygenase 2 (COX2), matrix metallopeptidase 9 (MMP9), vascular endothelial growth factor (VEGF), and CCND1 in a xenograft model.^37^ In a mouse model of CRC, 3′-HPSB has been shown to inhibit IL-6 signaling, suppress the expression of inflammatory enzymes, and reduce the activity of the β-catenin signaling pathway.^11^ In addition, another study using an HFD + DSS-induced colitis model demonstrated that 3′-HPSB significantly reduced IL-6 and MCP-1 levels, while also downregulating COX-2, PV-1, and p-STAT3 expression, suggesting a broader anti-inflammatory effect of 3′-HPSB under different pathological conditions.^12^

Despite these previously reported effects, the responses of Wnt/β-catenin-related proteins to PSB and 3′-HPSB in the present study differed between colonic and tumor tissues (Figures 6 and 7). Whereas several components of this pathway, including APC, GSK3β, β-catenin, and c-Myc, were altered by PSB and/or 3′-HPSB in colonic tissue, relatively limited changes were observed in established tumor tissue. One possible explanation is that established CRC lesions may exhibit more persistent and heterogeneous Wnt/β-catenin signaling, influenced by both tumor-intrinsic alterations and the tumor microenvironment, which may reduce their responsiveness to dietary interventions.^38,39^ In contrast, the surrounding colonic tissue may remain more responsive to modulation during earlier stages of disease development. This interpretation is also consistent with the preventive/protective design of the present study. However, the mechanisms underlying this tissue-specific difference were not directly investigated, and further studies are required to determine the factors responsible for the differential responses observed between colonic and tumor tissues.

Modulation of gut microbes may also contribute to the CRC suppression observed in our study. Our results indicate that PSB and 3′-HPSB increased the relative abundance of *Akkermansia* (Figure S7). Research has shown that oral administration of *Akkermansia* significantly reduced the number and size of tumors in AOM/DSS-induced mice compared to control mice. *Akkermansia* downregulated the expression of the aryl hydrocarbon receptor (*Ahr*), a potential upstream protein of β-catenin. This observation indicates that *Akkermansia* may inhibit β-catenin nuclear transcription by downregulating *Ahr* expression, further suppressing CRC development.^40^ In addition, alterations in gut microbiota composition were accompanied by changes in fecal SCFA levels in the present study. Previous experimental studies provide biological plausibility for a potential contribution of SCFAs to CRC attenuation. Tian et al. demonstrated that administration of an acetate, propionate, and butyrate mixture reduced tumor incidence and size, colonic inflammation, and epithelial proliferation in an AOM/DSS-induced colitis-associated CRC model.^41^ Similarly, Donohoe et al. demonstrated in a gnotobiotic mouse model that dietary fiber suppressed colorectal tumorigenesis in a microbiota- and butyrate-dependent manner, with butyrate accumulation associated with histone deacetylase inhibition, reduced cell proliferation, and increased apoptosis.^42^ These findings support the biological plausibility that the increased SCFA levels observed following PSB and 3′-HPSB supplementation may contribute to the attenuation of CRC progression. However, because the present study did not directly manipulate SCFAs or gut microbiota, their causal contribution to the observed protective effects cannot be established.

Another limitation of this study is the absence of a separate CRD-only control group. Therefore, the present design does not allow us to determine the independent effects of chronic circadian disruption on colonic physiology in otherwise healthy mice. Previous studies incorporating circadian disruption-only groups have shown that circadian disruption alone can induce circadian and molecular alterations in the colon without necessarily causing overt colonic histopathological injury under baseline conditions, whereas intestinal pathological changes become substantially more pronounced following DSS-induced inflammatory challenge.^43,44^ These findings provide relevant context for the present study, in which chronic jet lag was incorporated as an additional disease-aggravating condition rather than as an independent intestinal disease model. Nevertheless, a dedicated CRD-only group would be required to determine whether similar effects occur under our chronic jet lag protocol and to distinguish the independent effects of circadian disruption from its interaction with AOM/DSS-induced tumorigenesis.

The physiological relevance of the chronic jet lag protocol should also be considered when interpreting the present findings. Repeated phase shifts of the light-dark cycle are established experimental approaches for inducing circadian misalignment in rodents and have been used to model aspects of circadian disruption associated with jet lag and rotating shift work.^45,46^ Notably, a previous chronic phase-advance study using a protocol similar to ours (6-h advance every 4 days) demonstrated alterations in locomotor, cardiovascular, thermoregulatory, and peripheral molecular clock rhythms.^47^ In addition, studies of 6-h phase advances have shown that resynchronization may require several days and can occur at different rates across central and peripheral circadian oscillators.^48^ Thus, the repeated 6-h advances used in the present study provide an experimental model of recurrent circadian misalignment. However, this controlled phase-shift paradigm does not fully reproduce the heterogeneous patterns of circadian disruption experienced by humans, which can vary in shift timing, frequency, direction, light exposure, sleep-wake behavior, and individual adaptation. Therefore, the present model should be regarded as an experimental approximation of sustained circadian misalignment rather than a direct replication of typical human shift-work or travel schedules.

In addition, although PSB and 3′-HPSB supplementation was accompanied by changes in several circadian clock-related proteins, the present experimental design does not establish that modulation of circadian rhythm is required for, or causally mediates, their protective effects against CRC progression. Dedicated experiments involving direct manipulation of circadian clock function, such as genetic manipulation of BMAL1, will be required to determine this relationship. Although β-catenin protein expression and representative immunofluorescence staining in intestinal epithelial regions were evaluated, nuclear/cytoplasmic β-catenin distribution was not quantitatively assessed. Therefore, the present findings do not directly establish changes in β-catenin nuclear localization, and future studies using nuclear/cytoplasmic fractionation or quantitative high-resolution localization analysis will be required to more directly evaluate canonical Wnt/β-catenin pathway activation.

Similarly, although alterations in gut microbiota composition and SCFA levels were observed alongside the protective effects of PSB and 3′-HPSB, the present study does not establish their causal roles in CRC attenuation. Future studies incorporating direct SCFA supplementation or receptor-related analyses, together with gut microbiota depletion, recolonization, or fecal microbiota transplantation, will be required to determine the mechanistic contributions of SCFAs and gut microbiota to these protective effects.

Intestinal barrier function was also not directly evaluated in the present study. Previous studies have shown that PSB can attenuate intestinal barrier dysfunction and preserve tight junction-related proteins in experimental models of intestinal injury and metabolic inflammation.^49,50^ In addition, our previous study examining 3′-HPSB in DSS-induced colitis reported alterations related to intestinal epithelial integrity together with changes in inflammatory responses and gut microbiota composition.^13^ However, these findings from other experimental models cannot establish whether preservation of intestinal barrier integrity contributed to the protective effects of PSB or 3′-HPSB in the present AOM/DSS chronic jet lag model. Therefore, whether intestinal barrier function contributes to the associations among PSB/3′-HPSB supplementation, gut microbiota alterations, and CRC progression remains unknown. Future studies incorporating direct intestinal permeability assays and evaluation of tight junction proteins will be required to address this possibility. The relative contributions of PSB, 3′-HPSB, and their downstream metabolites in vivo also remain to be fully elucidated and will require further pharmacokinetic and metabolomic analyses.

Furthermore, PSB and 3′-HPSB supplementation was initiated 1 week before AOM/DSS administration as a pretreatment, reflecting a preventive/protective protocol rather than a therapeutic design. Therefore, the present findings should primarily be interpreted in the context of CRC prevention/protection, and additional studies employing postinsult therapeutic protocols will be required to evaluate the therapeutic efficacy of PSB and 3′-HPSB.

The present study did not include a pharmacological positive control. Although the experimental design allowed evaluation of the effects of PSB and 3′-HPSB relative to the untreated JAD group, the absence of a positive control limits direct comparison of their protective efficacy with an established intervention. Future studies incorporating an appropriate positive control, such as 5-aminosalicylic acid (5-ASA), would provide a useful benchmark for evaluating the relative efficacy of these dietary compounds. Future studies may explore the inclusion of validated oral compounds or alternative strategies as positive controls to benchmark the preventive efficacy of PSB and 3′-HPSB.

This study tested a single fixed dose of each compound. The 0.025% dietary concentration of 3′-HPSB was selected based on our previous studies demonstrating its protective effects in mouse models of intestinal inflammation,^11–13^ while PSB was administered at the same concentration to enable direct comparison between the two compounds under equivalent dietary conditions. Although the selected concentrations were supported by previous efficacy and safety evidence, the use of a single dose in the present study does not allow characterization of dose–response relationships or determination of the optimal effective dose and safety range. Future studies incorporating multiple dose levels will therefore be required to establish dose–response relationships and further evaluate dose-dependent effects on CRC-related outcomes and associated molecular changes.

Finally, the clinical translation of pterostilbene (PSB) and 3′-hydroxypterostilbene (3′-HPSB) requires further evaluation. Based on the estimated dietary exposure of 56.8 mg/kg body weight/day in the present study, body surface area scaling yields an approximate human equivalent dose of 4.6 mg/kg/day (≈280 mg/day for a 60-kg adult). This estimate is slightly higher than the PSB doses previously evaluated in human clinical studies and reported to be generally safe and well tolerated (50–125 mg twice daily,^51^ up to 250 mg/day^52^). In addition, a 28-day toxicity study in mice reported no mortality or notable biochemical or histopathological adverse effects following oral PSB administration at doses up to 3000 mg/kg body weight/day, providing additional preclinical safety context for the substantially lower PSB exposure used in the present study.^53^ For 3′-HPSB, a 90-day repeated oral toxicity study conducted in Sprague–Dawley rats reported no observed adverse effects up to 200 mg/kg/day.^54^ Using standard body surface area scaling, this corresponds to a mouse equivalent dose of approximately 400 mg/kg/day, which is approximately 7-fold higher than the estimated 3′-HPSB exposure of 56.8 mg/kg/day in the present study. This comparison provides supportive preclinical safety context but should not be interpreted as establishing a definitive safety margin. Overall, these findings provide preliminary safety context for the doses used in the present study. However, additional studies are needed to establish the long-term safety, pharmacokinetics, and optimal dosing strategies of PSB and 3′-HPSB for future clinical applications.

Pharmacokinetic factors should also be considered when extrapolating these dietary doses to humans.^55^ Although PSB exhibits relatively favorable oral bioavailability compared with resveratrol, its systemic exposure can still be influenced by physicochemical and formulation-related factors, including its low aqueous solubility.^56^ In addition, PSB undergoes multiple metabolic transformations, including formation of the pharmacologically active metabolite 3′-HPSB, and the kinetics of this metabolic conversion differ substantially among species.^57^ Pharmacokinetic information on 3′-HPSB remains more limited; however, a preliminary study in rats demonstrated rapid disposition following intravenous administration, with glucuronidation representing a major metabolic pathway.^58^ Therefore, body surface area-based human equivalent dose estimates should be regarded as approximate dose-scaling references rather than direct predictors of equivalent systemic or intestinal exposure in humans. Further pharmacokinetic studies, particularly following oral administration, will be required to characterize the absorption, metabolism, and bioavailability of PSB and 3′-HPSB and to establish clinically relevant dosing strategies.

To improve intestinal targeting and bioavailability, future studies may explore encapsulation strategies, such as microsphere carriers or other advanced drug delivery systems.^59^ Additionally, combining PSB/3′-HPSB with low-dose nonsteroidal anti-inflammatory drugs (NSAIDs), such as low-dose aspirin, could enhance preventive efficacy while minimizing potential adverse effects.^60^ These approaches, together with further pharmacokinetic and long-term safety studies, will help establish optimized dosing strategies. Moreover, the design of early phase clinical trials to assess safety, tolerability, and preliminary preventive effects in at-risk populations will be critical for translating these preclinical findings into clinical application.

In summary, dietary supplementation with PSB and 3′-HPSB attenuated CRC progression exacerbated by circadian rhythm disruption. These protective effects were accompanied by reduced inflammatory responses, alterations in circadian clock-related proteins and Wnt/β-catenin signaling-related proteins, and reduced intestinal epithelial cell proliferation. Additionally, PSB and 3′-HPSB supplementation was associated with alterations in gut microbiota composition and increased levels of several SCFAs, which may partially contribute to the observed protective effects. Given the pretreatment design of the present study, these effects should primarily be interpreted as preventive/protective rather than therapeutic. Collectively, these findings support the potential of PSB and 3′-HPSB as dietary interventions for CRC prevention, while further studies are required to determine the causal contributions of gut microbiota and SCFAs and to clarify the regulatory relationship between core clock genes and Wnt/β-catenin signaling.

## Supplementary Material



## Data Availability

The data that support the findings of this study are available in the Supporting Information of this article.

## Notes

Experimental design and chronic circadian rhythm disruption model (Figure S1); representative immunofluorescence staining of *K*_i_-67 in colorectal tumor tissues (Figure S2); representative immunofluorescence staining of caspase-3 in colorectal tumor tissues (Figure S3); representative immunofluorescence staining of β-catenin in intestinal epithelial regions (Figure S4); Mantel test correlation analysis (Figure S5); gut microbiota α-diversity analysis (Figure S6); gut microbial composition analysis (Figure S7) (PDF)

## Abbreviations Used

ALT: alanine aminotransferase
AOM: azoxymethane
AST: aspartate aminotransferase
AUC: area under the curve
BMAL1: basic helix–loop–helix ARNT-like 1
CLOCK: circadian locomotor output cycles kaput
c-Myc: myelocytomatosis oncogene
CRC: colorectal cancer
CRD: circadian rhythm disorder
CRY1: cryptochrome circadian regulator 1
DAI: disease activity index
DSS: dextran sulfate sodium
3′-HPSB: 3′-hydroxypterostilbene
IL-1β: interleukin-1β
MCP-1: monocyte chemoattractant protein-1
LRP6: low-density lipoprotein receptor-related protein 6
PCoA: principal coordinates analysis
PER: period circadian clock
PLS-DA: partial least-squares discriminant analysis
PSB: pterostilbene
REV-ERBα: reverse eukaryotic regulatory binding protein alpha
SCFA: short-chain fatty acids
TNF-α: tumor necrosis factor-alpha
Wnt-3A: wingless-type MMTV integration site family member 3A
