# Supplementary material for: Pterostilbene and 3′-Hydroxypterostilbene Alleviate AOM/DSS-Induced and Chronic Jet Lag-Exacerbated Colon Cancer
Source: J Agric Food Chem. 2026 Oct 26;74(43):33963–78. doi: 10.1021/acs.jafc.6c09704 (PMC13741832; doi:10.1021/acs.jafc.6c09704)
Supplement: Supplementary file 1 [file jf6c09704_si_001.pdf]

Supporting information

Pterostilbene and 3′-hydroxypterostilbene alleviate AOM/DSS-induced and chronic jet lag-exacerbated colon cancer

Yen-Chun Koh[ʔ], Yen-Tung Chen[†], Pin-Yu Ho[†], Wei-Sheng Lin[‡], Kai-Yu Hsu[†], Anju Majeed[§], Chi-Tang Ho[||], Min-Hsiung Pan [†, ¶, @, *]

ʔ Department of Seafood Science, National Kaohsiung University of Science and Technology, 81157, Kaohsiung, Taiwan

[†] Institute of Food Sciences and Technology, National Taiwan University, 10617 Taipei, Taiwan

[‡] Department of Food Science, National Quemoy University, 89250 Quemoy, Taiwan

[§] Sami-Sabinsa Group Limited, Bengaluru, 560058 Karnataka, India

[||] Department of Food Science, Rutgers University, New Brunswick, 08901 New Jersey, USA

[¶] Department of Medical Research, China Medical University Hospital, China Medical University, 40402 Taichung City, Taiwan

[@] Department of Health and Nutrition Biotechnology, Asia University, 41354 Taichung City, Taiwan

*** Please send all correspondence to:**

Dr. Min-Hsiung Pan

Institute of Food Science and Technology,

National Taiwan University,

No. 1, Section 4, Roosevelt Road, Taipei 10617, Taiwan.

Tel. no. +886-2-33664133

Fax. no. +886-2-33661771

E-mail: mhpan@ntu.edu.tw

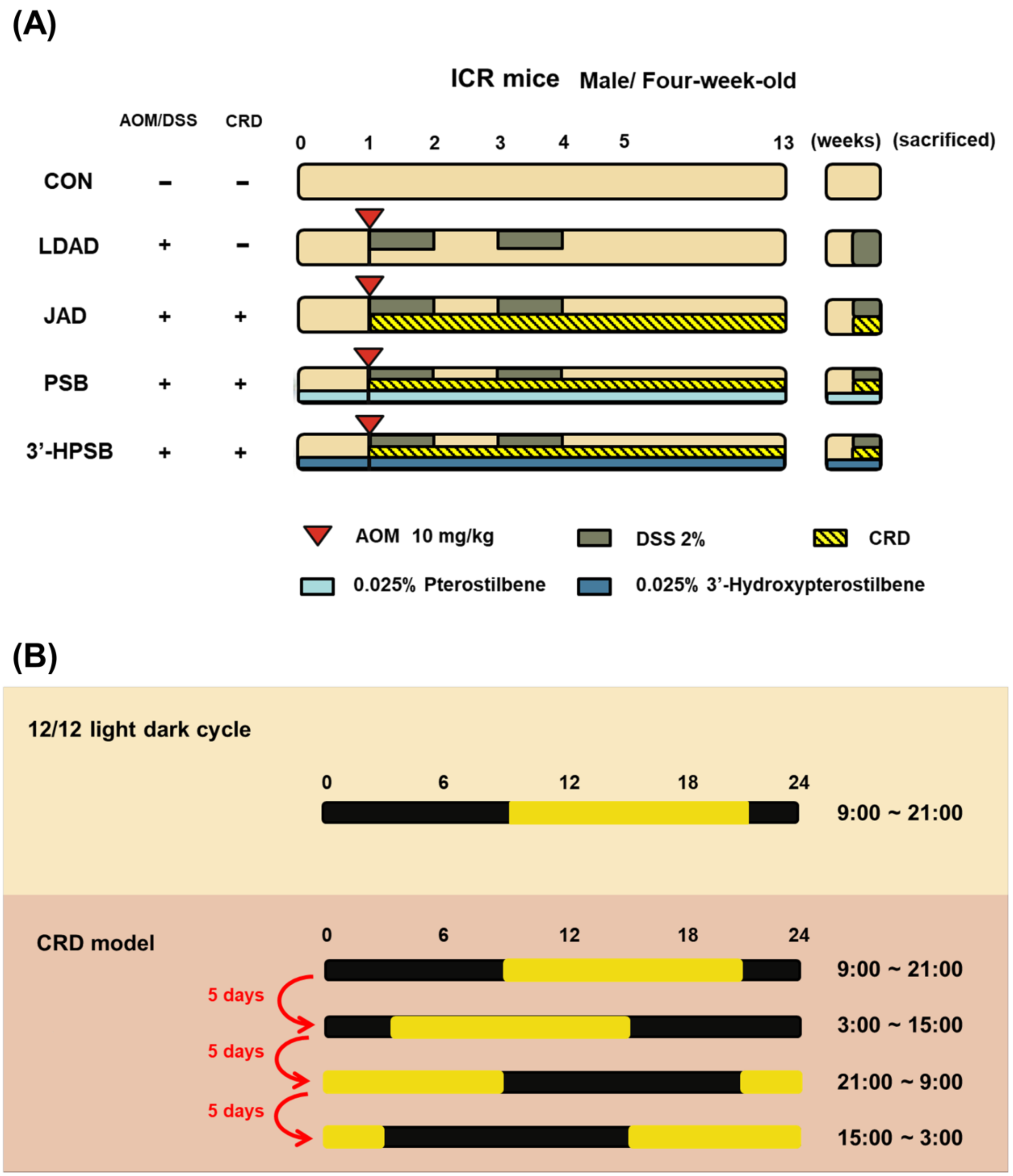

Figure S1. (A) Experimental design and (B) the model used to disrupt circadian rhythm.

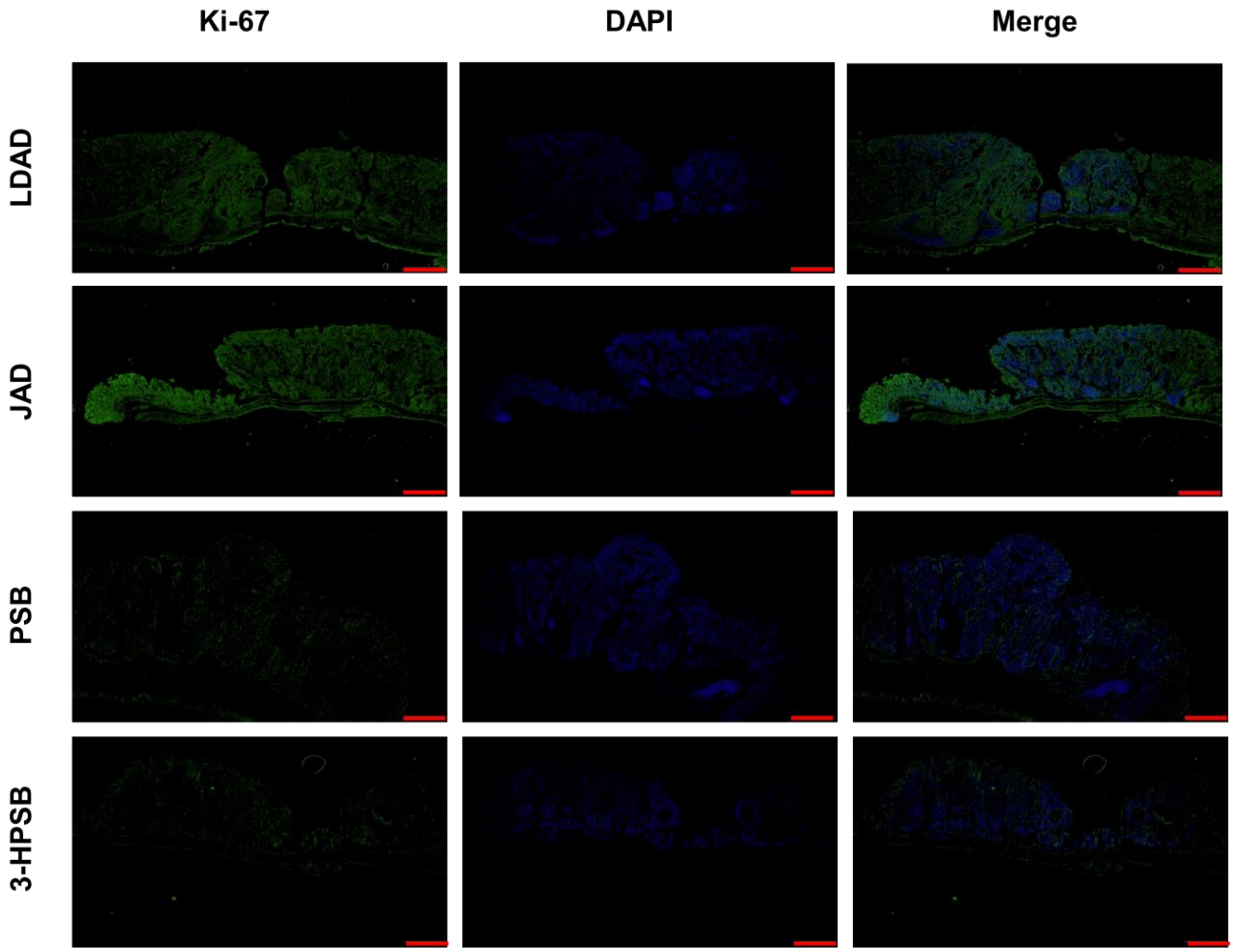

Figure S2. Representative immunofluorescence staining of Ki-67 in colorectal tumor tissues.

Representative immunofluorescence images of Ki-67 in tumor tissues from the LDAD, JAD, PSB, and 3′-HPSB groups are shown. Tissue sections were incubated with Ki-67 (D3B5) Rabbit Monoclonal Antibody (1:100; #9129, Cell Signaling Technology), followed by a fluorescent secondary antibody (1:1000; GeneTex). Nuclei were counterstained with DAPI. Ki-67 staining is shown in green and nuclei in blue. Images are representative of the staining patterns observed in each experimental group. Images were acquired at 100× magnification. Scale bars = 500 μm.

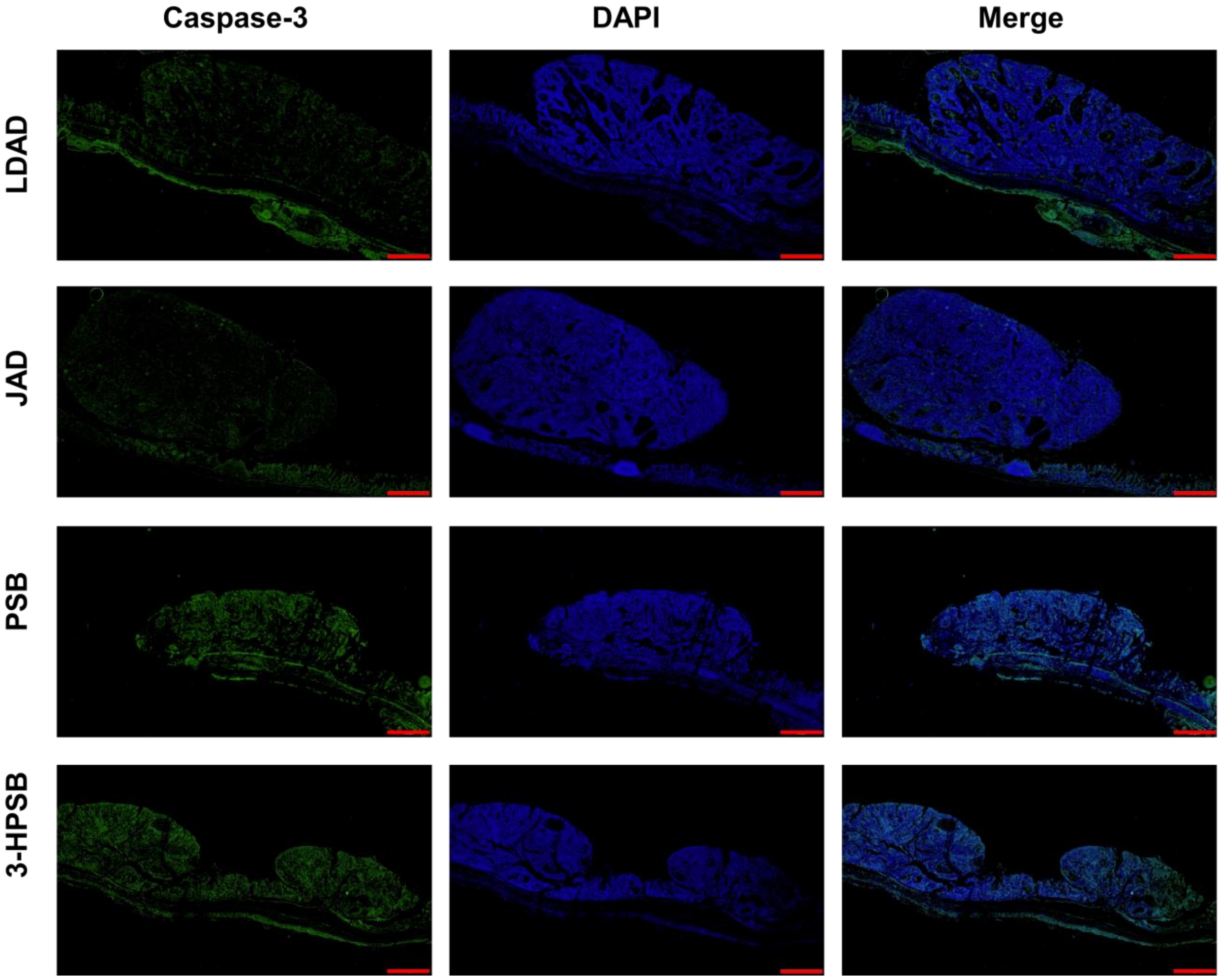

Figure S3. Representative immunofluorescence staining of caspase-3 in colorectal tumor tissues.

Representative immunofluorescence images of caspase-3 in tumor tissues from the LDAD, JAD, PSB, and 3′-HPSB groups are shown. Tissue sections were incubated with Caspase-3 Antibody (1:100; #9662, Cell Signaling Technology), followed by a fluorescent secondary antibody (1:1000; GeneTex). Nuclei were counterstained with DAPI. Caspase-3 immunoreactivity is shown in green and nuclei in blue. Images are representative of the staining patterns observed in each experimental group. Images were acquired at 100× magnification. Scale bars = 500 μm.

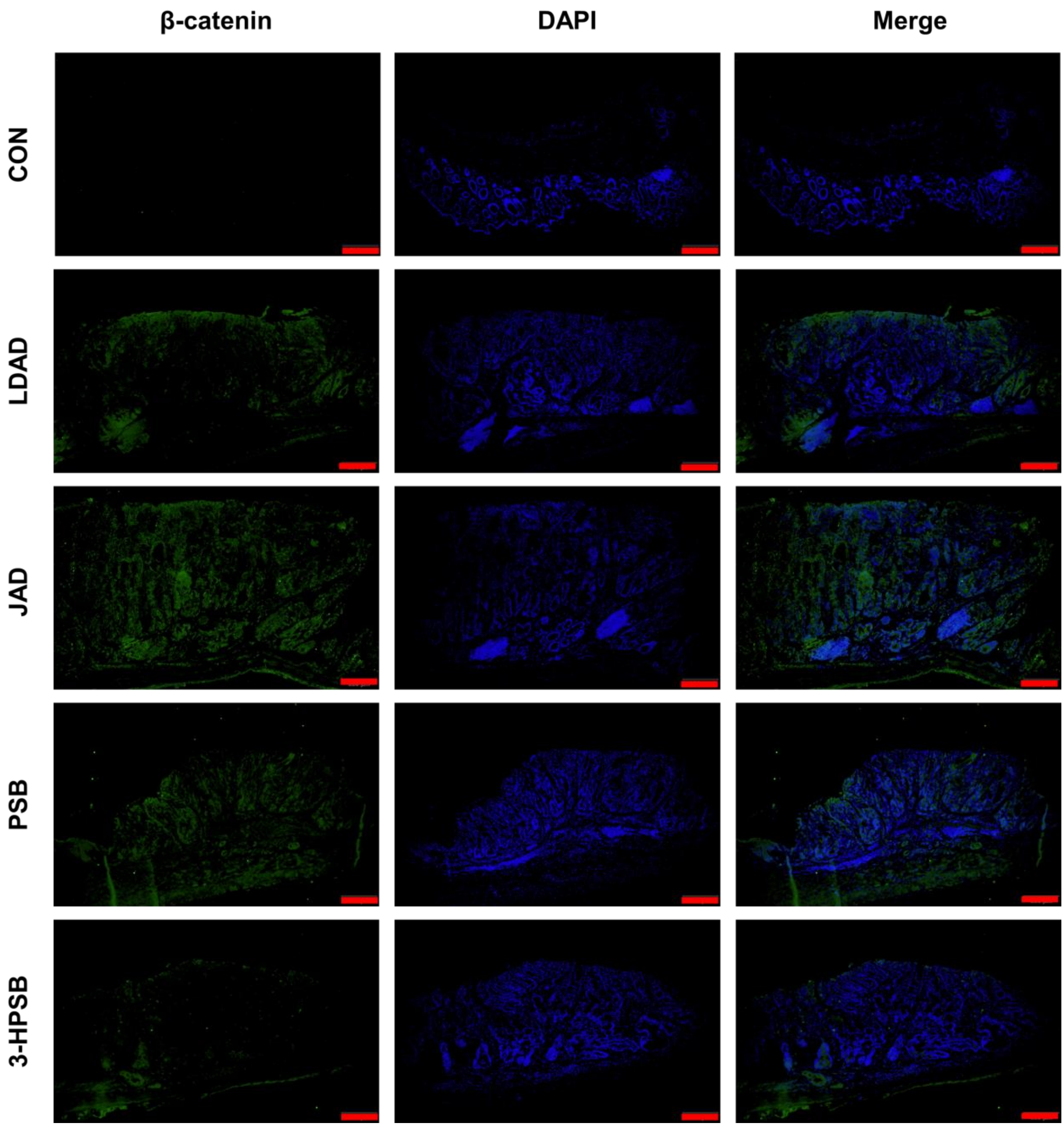

Figure S4. Representative immunofluorescence staining of β-catenin in intestinal epithelial regions.

Representative immunofluorescence images of β-catenin in intestinal epithelial regions from the CON, LDAD, JAD, PSB, and 3′-HPSB groups are shown. Tissue sections were incubated with β-catenin antibody, followed by a fluorescent secondary antibody. Nuclei were counterstained with DAPI. β-Catenin immunoreactivity is shown in green and nuclei in blue. Images were selected to represent intestinal epithelial regions corresponding to the tissue evaluated by Western blot

analysis and are representative of the staining patterns observed in each experimental group. Images were acquired at 200× magnification. Scale bars = 200 μm.

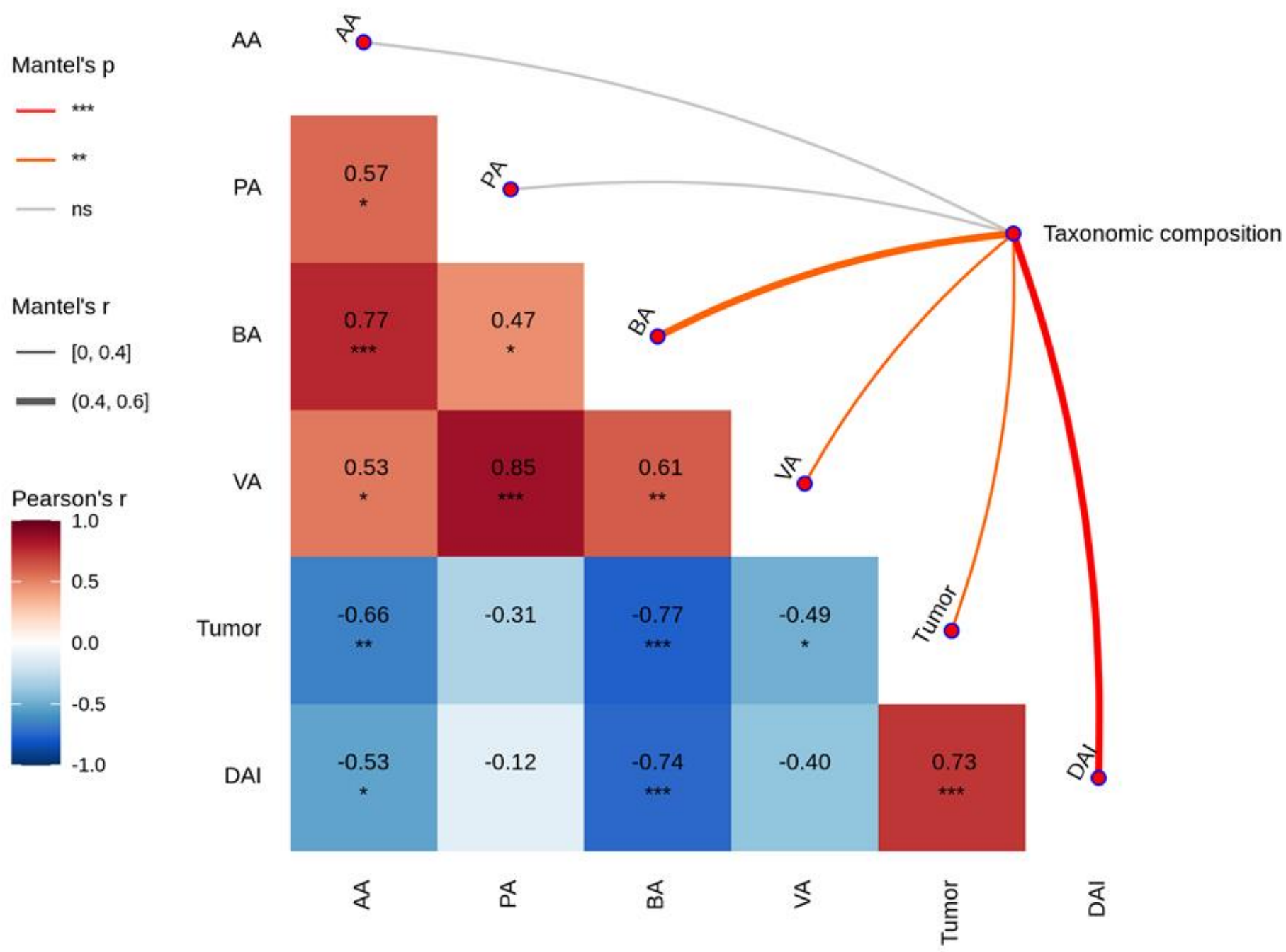

Figure S5. Mantel test correlation plot.

PA = propionic acid; VA = valeric acid; BA = butyric acid; DAI = disease activity index.

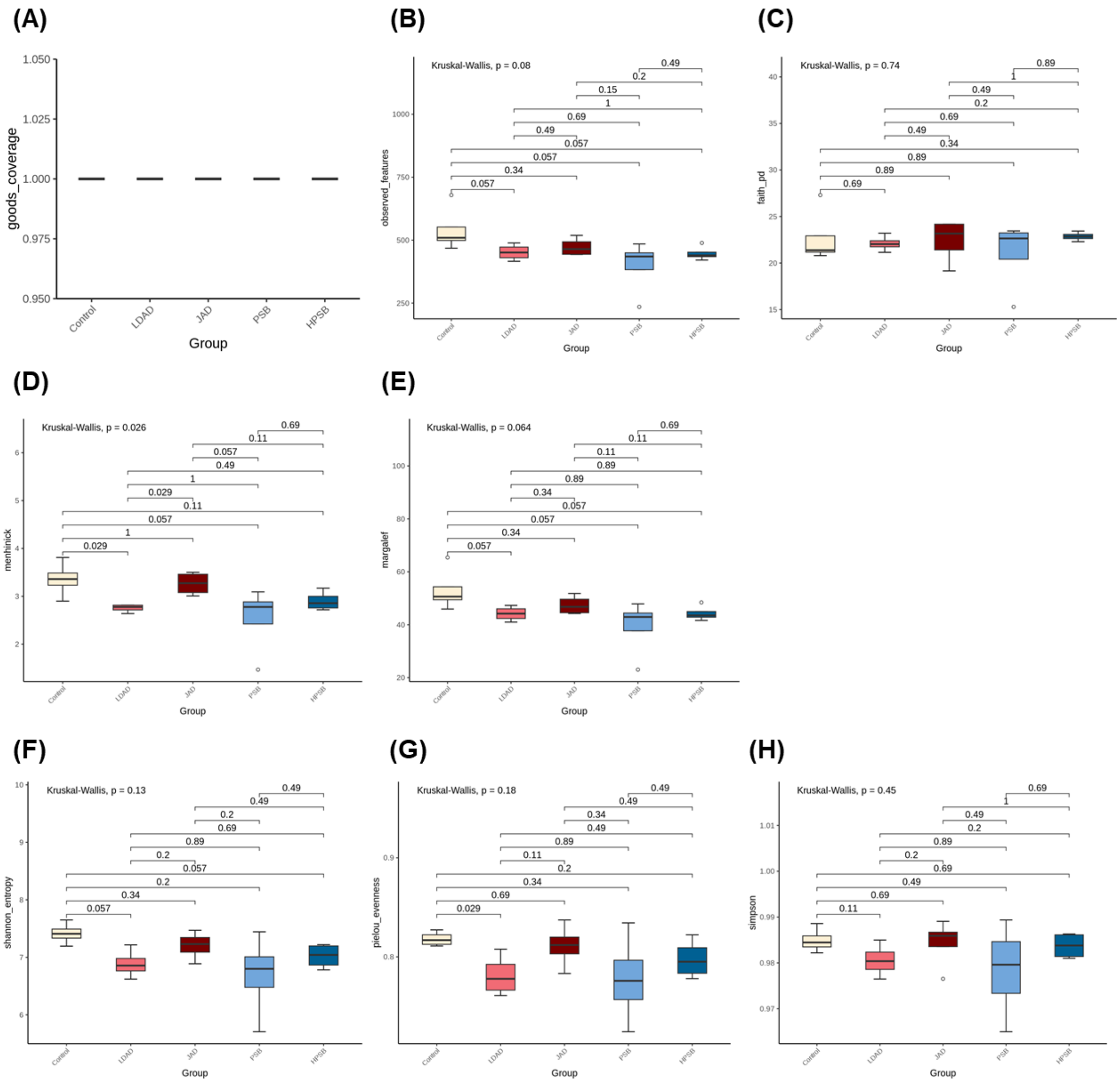

Figure S6. Chronic jet lag and AOM/DSS treatment did not significantly alter gut microbiota α-diversity.

α-diversity indices, including (A) Goods coverage, (B) Observed features, (C) Faith's phylogenetic diversity (Faith's PD), (D) Menhinick richness index, Margalef richness index, Shannon entropy, Pielou's evenness, and Simpson index, were compared across the Control, LDAD, JAD, PSB, and HPSB groups. Statistical analysis was performed using the Kruskal-Wallis test followed by pairwise comparisons. No significant differences were observed between groups ($p > 0.05$ for all indices), indicating that chronic jet lag and AOM/DSS treatment did not significantly affect the overall gut microbial α-diversity.

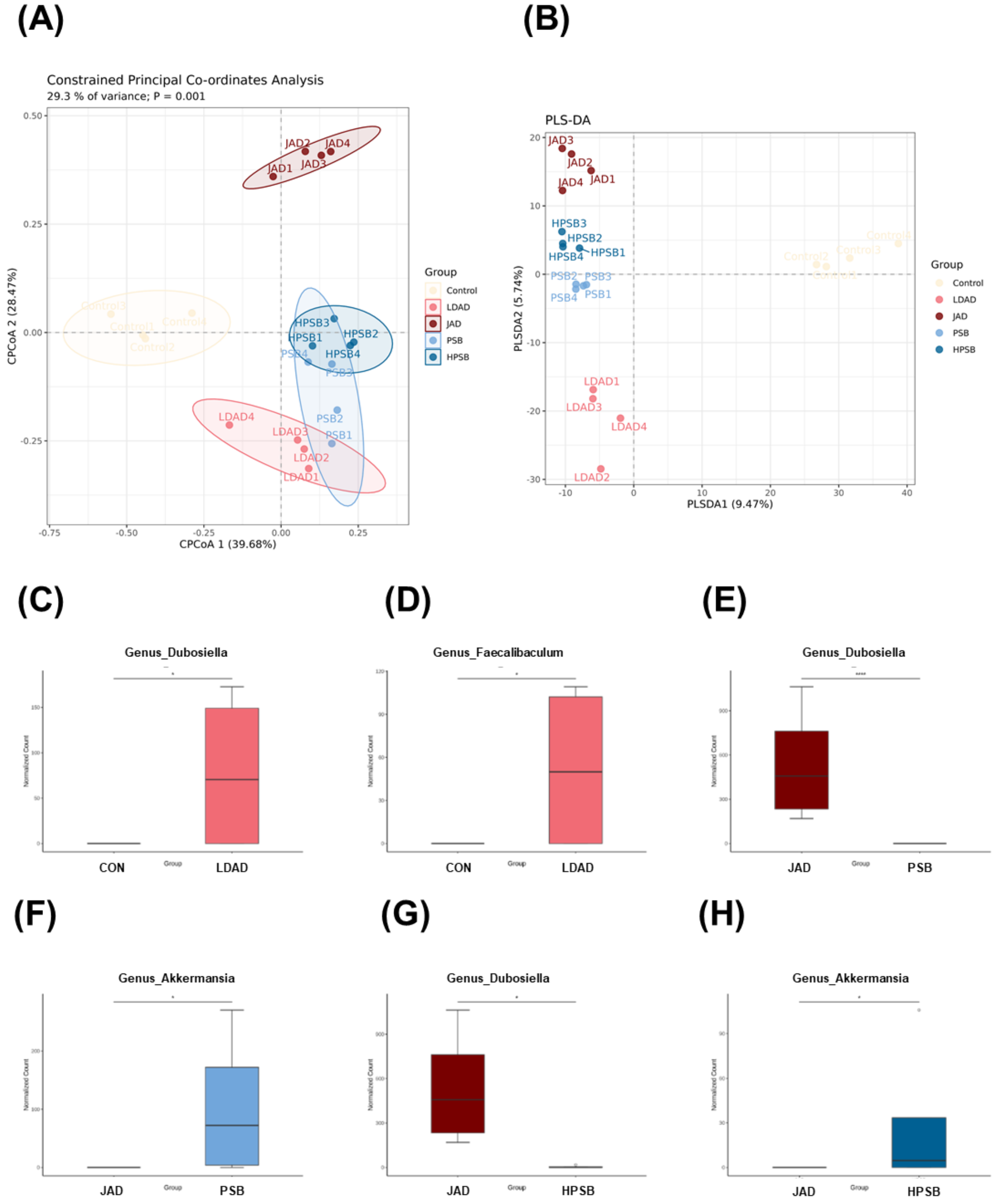

Figure S7. Gut microbial composition in each group.

(**A**) PCoA plot. (**B**) PLS-DA plot. (**C-H**) Metagenomic analysis showing differences in relative abundance between the compared groups at the genus level.

Table S1. List of primary antibodies and ELISA kits

	**Brand**
Primary antibody	
c-Myc	Proteintech (IL, USA)
Glycogen synthase kinase 3 beta (GSK3β)	
Cryptochrome circadian regulator 1 (CRY1)	
REV-ERBα, axin 1 (AXIN1)	Cell Signalling Technology (MA, USA)
β-catenin	
Total/phosphorylated LDL receptor-related protein 6 (t-/p-LRP6)	
APC	ABclonal Biotech Co., Ltd. (China)
Wnt family member 3A (WNT3A)	
CLOCK	Abcam Plc (Cambridge, UK)
BMAL1	
Glyceraldehyde-3-phosphate dehydrogenase (GAPDH)	
PER2	Sigma-Aldrich (MA, USA)
Actin beta (ACTB/β-actin)	
Elisa kit	
Tumor necrosis factor-α (TNF-α, catalog #88-7324-88)	Invitrogen, CA, US
IL-6 (catalog #88-7064-88)	
IL-1β catalog #88-7013A-88)	
Chemokine monocyte chemoattractant protein-1 (MCP-1, catalog #88-7391-22)	

Methods

DAI measurement

The indicators and the criteria for DAI scoring is provided in supplementary material. The DAI consists of four indicators, each scored on a 0-4 scale based on severity: (1) Body weight loss: 0 points (<1%), 1 point (1%-5%), 2 points (5%-10%), 3 points (10%-20%), and 4 points (>20%); (2) Stool consistency: 0 points (normal), 1 point (soft), 2 points (semi-solid), 3 points (loose stool), and 4 points (diarrhea); (3) Fecal occult blood test: 0 points (no color change), 1 point (green), 2 points (blue-green), 3 points (blue), and 4 points (dark blue); (4) Anus appearance: 0 points (normal), 1 point (rectal prolapse), 2 points (red, swollen), 3 points (bloody stool or signs of past bleeding), and 4 points (fresh blood or tumor).

Western blot procedures

Tissue samples from the scraped colon mucosa, liver, and tumors were homogenized in cold lysis buffer and vortexed on ice for 1 hour, followed by centrifugation at 14,000 × g at 4 °C for 30 minutes. The protein concentration in the supernatant was determined using a Bio-Rad protein assay kit (Bio-Rad, Hercules, CA, USA). Equal amounts of protein (25 µg per sample) were separated by 10% SDS-PAGE and transferred onto polyvinylidene difluoride (PVDF) membranes. Membranes were blocked with 5% bovine serum albumin (BSA) for 1 hour at room temperature and incubated overnight at 4 °C with primary antibodies against the target proteins, followed by incubation with horseradish peroxidase (HRP)-conjugated secondary antibodies for 1 hour at room temperature. Protein bands were visualized using an enhanced chemiluminescence (ECL) detection kit and imaged using the eBLOT wet blotting transfer system. Western blot band intensities were quantified using ImageJ software (National Institutes of Health, Bethesda, MD, USA). Target protein expression levels were first normalized to the corresponding housekeeping proteins (β-actin or GAPDH) to correct for loading variability.

Fecal SCFA analysis

First, 3–4 stainless steel beads were placed in a fecal homogenization tube, and 0.1 g of colon feces was added along with 0.5 mL of a 0.5% phosphoric acid solution. Next, the mixture was homogenized using two cycles of 7 m/s for 15 seconds. Then, a tabletop centrifuge was used to reduce foam. Next, 0.75 mL of ethyl acetate was added, and the mixture was re-homogenized under the same conditions. Then, the sample was

centrifuged at 18,000 rpm and 4°C for 10 minutes, and 0.2 mL of the supernatant was transferred to a separate 1.5 mL centrifuge tube. Next, 0.8 mL of 625 μM 4-methylvaleric acid was added as an internal standard and mixed thoroughly. Then, the solution was filtered through a 0.22 μm nylon syringe filter into a vial for subsequent analysis using a DB-WAXetr column with the following settings: fixed flow rate = 1 mL/min, mode = splitless, column temperature = 250°C, auxiliary gas temperature = 280°C, ion source temperature = 230°C, quadrupole temperature = 150°C, mass-to-charge ratio (m/z) detection range = 30–250, and solvent retention time = 3.5 minutes. The oven temperature was adjusted as follows: 90°C at 0 minutes, increasing to 150°C at 4 minutes (at 15°C/min), 170°C at 8 minutes (at 5°C/min), and 250°C at 12 minutes (at 20°C/min), holding this temperature until 14 minutes.

Gut microbiota analysis

Genomic DNA was extracted and purified using the innuPREP Stool DNA Isolation Kit (Analytik Jena, Germany) following the manufacturer's instructions. Primers containing a 5' buffer sequence (GCATC) and a 5' phosphate modification were designed to amplify the full-length 16S rRNA gene. High-fidelity (HiFi) reads with a predicted accuracy of Phred score ≥ 30 were generated on the PacBio Sequel IIe platform (Pacific Biosciences, Menlo Park, CA, USA) in circular consensus sequencing (CCS) mode. On average, 28,026 ± 609 raw HiFi reads per sample were obtained (range: 23,500–34,700 reads). After primer trimming and quality filtering, an average of 25,160 ± 651 filtered reads per sample were retained. The processed reads were analyzed using the DADA2 pipeline (version 1.20) to resolve amplicon sequence variants (ASVs) at single-nucleotide resolution. The DADA2 workflow included quality filtering, dereplication, dataset-specific error model learning, ASV inference, and chimera removal. Trimming and filtering were performed with a maximum of two expected errors per read (maxEE = 2) to ensure high-quality sequence retention. After denoising and chimera removal, an average of 24,506 ± 652 high-quality non-chimeric reads per sample were retained for downstream taxonomic profiling and diversity analyses.

Alpha diversity indices, including the Shannon index, Faith's phylogenetic diversity (Faith PD), Menhinick index, Margalef index, and Good's coverage, were calculated using QIIME2. Good's coverage exceeded 99% across all samples, confirming sufficient sequencing depth. Differences in alpha diversity between groups were analyzed using the Kruskal-Wallis test. To identify differentially abundant bacterial taxa, linear

discriminant analysis effect size (LEfSe) analysis was performed. The Kruskal-Wallis test followed by the Wilcoxon rank-sum test was applied to detect significant differences in relative abundance between groups. The LDA score threshold was set to log10 > 3.0 to define significant biomarkers.
